# Interacting innovation processes

**DOI:** 10.1038/s41598-023-43967-1

**Published:** 2023-10-11

**Authors:** Giacomo Aletti, Irene Crimaldi, Andrea Ghiglietti

**Affiliations:** 1 ADAMSS Center, Università degli Studi di Milano, Milan, Italy; 2 IMT School for Advanced Studies Lucca, Lucca, Italy; 3 Università degli Studi di Milano-Bicocca, Milan, Italy

**Keywords:** Mathematics and computing, Applied mathematics, Computational science, Statistics

## Abstract

In this work, we introduce a general model for a collection of innovation processes in order to model and analyze the *interaction* among them. We provide theoretical results, analytically proven, and we show how the proposed model fits the behaviors observed in some real data sets (from *Reddit* and *Gutenberg*). It is worth mentioning that the given applications are only examples of the potentialities of the proposed model and related results: due to its abstractness and generality, it can be applied to many interacting innovation processes.

## Introduction

Analyzing the *innovation* process, that is the underlying mechanisms through which novelties emerge, diffuse and trigger further novelties is definitely of primary importance in many areas (biology, linguistics, social science and others^1–12^). We can define *novelties * (or *innovations*) as the first time occurrences of some event. A widely used mathematical object that models an innovation process is an *urn model with infinitely many colors*, also known as *species sampling sequence*^13–15^. Let $C_1$ be the first observed color, then, given the colors $C_1,\dots , C_t$ of the first *t* extractions, the color of the $(t+1)$th extracted ball is new (i.e. not already drawn in the previous extractions) with a probability $Z^*_t$ which is a function of $C_1,\dots ,C_t$ (sometimes called “birth probability”) and it is equal to the already observed color *c* with probability $P_{c,t}=\sum _{n=1}^t Q_{n,t}I_{\{C_n=c\}}$, where $Q_{n,t}$ is a function of $C_1,\dots ,C_t$. The quantities $Z^*_t$ and $Q_{n,t}$ specify the model: precisely, $Z^*_t$ describes the probability of having a new color (that is a novelty) at time-step $t+1$ and $Q_{n,t}$ is the weight at time-step *t* associated to extraction *n*, with $1\le n\le t$, so that the probability of having at time-step $t+1$ the “old” color *c* is proportional to the total weight at time-step *t* associated to that color (a *reinforcement mechanism*, sometimes called “weighted preferential attachment” principle). Note that the number of possible colors is not fixed a priori, but new colors continuously enter the system. We can see the urn with infinitely many colors as the space of possibilities, while the sequence of extracted balls with their colors represents the history which has been actually realized.

The *Blackwell–MacQueen urn* scheme^14,16^ provides the most famous example of innovation process. According to this model, at time-step $t+1$ a new color is observed with probability given by a deterministic function of *t*, that is $Z^*_t=z^*(t)=\theta /(\theta +t)$, where $\theta >0$, and an old color is observed with a probability proportional to the number $K_{c,t}$ of times that color was extracted in the previous extractions: $Q_{n,t}=q_n(t)=1/(\theta +t)$, i.e. $P_{c,t}=K_{c,t}/(\theta +t)$. This is the “simple” preferential attachment rule, also called “popularity” principle. This urn model is also known as Dirichlet process^17^ or as Hoppe’s model^18^ and, in terms of random partitions, it corresponds to the so called *Chinese restaurant process*^19^. Afterwards, it has been extended introducing an additional parameter and it has been called *Poisson-Dirichlet model*^19–22^. More precisely, for the Poisson–Dirichlet model, we have

1 $$\begin{aligned}{}&Z^*_t=\frac{\theta +\gamma D_t}{\theta + t}, \qquad Q_{n,t}=\frac{1-\gamma /K_{C_n,t}}{\theta + t},\\&\quad \text{ and } \text{ so } \quad P_{c,t}= \frac{K_{c,t}-\gamma }{\theta + t}, \end{aligned}$$

where $0\le \gamma <1$, $\theta >-\gamma$ and $D_t$ denotes the number of distinct extracted colors until time-step *t*. From an applicative point of view, as an innovation process, the Poisson-Dirichlet process has the merit to reproduce in many cases the correct basic statistics, namely the Heaps’^23,24^ and the (generalized) Zipf’s laws^25–27^, which quantify, respectively, the rate at which new elements appear and the frequency distribution of the elements. In particular, the *Heaps’ law* states that the number $D_t$ of distinct observed elements (i.e. colors, according to the metaphor of the urn) when the system consists of *t* elements (i.e. after *t* extractions from the urn) follows a power law with an exponent smaller than or equal to 1 and, for the Poisson-Dirichlet model, we have $D_t\propto t^{\gamma }$ for $0<\gamma <1$ (while $D_t\propto \ln (t)$ for $\gamma =0$).

Recently, a new model, called *urn with triggering*, that includes the Poisson-Dirichlet process as a particular case, have been introduced and studied^28–31^. This model is based on Kauffman’s principle of the adjacent possible^32^: indeed, the model starts with an urn with a finite number of balls with distinct colors and, whenever a color is extracted for the first time, a set of balls with new colors is added to the urn. This represents Kauffman’s idea that, when a novelty occurs, it triggers further potential novelties. In particular, the urn with triggering has the merit to provide a very clear representation of the evolution dynamics of the Poisson–Dirichlet process. An urn initially contains $N_0>0$ distinct balls of different colors. Then, at each time step $t+1$, a ball is drawn at random from the urn and

- if the color of the extracted ball is new, i.e. it was not been extracted in the previous extractions, then we replace the extracted ball by ${\widehat{\rho }}$ balls of the same color as the extracted ball plus $(\nu +1)$ balls of distinct new colors, i.e. not already present in the urn;
- if the color of the extracted ball is old, i.e. it has been already extracted in the previous extractions, we replace the extracted ball by $(1+\rho )$ balls of the same color as the extracted one.

It easy to verify that, when the *balance condition* ${\widehat{\rho }}+\nu =\rho$ is satisfied (this means that at each time-step the number of balls added to the urn is always $\rho$, regardless of the outcome of the extraction), the above updating rule gives rise to the above probabilities (1), taking $\rho >\nu \ge 0$, $\theta =N_0/\rho$ and $\gamma =\nu /\rho$.

Given the importance of understanding how different innovation processes affect each other, this work aims at introducing and analyzing a model for a finite network of innovation processes. In the proposed model, for each node *h*, the probability of observing a new or an old item depends, not only on the path of observations recorded for *h* itself, but also on the outcomes registered for the other nodes $j\ne h$. More precisely, we introduce a system of *N* urns with triggering that interact each other as follows:

(i) the probability of *exploitation* of an old item *c* by node *h*, i.e. the probability of extracting from urn *h* a color *c* already drawn in the past from an urn of the system (not necessarily from *h* itself), has an increasing dependence not only on the number of times *c* has been observed in node *h* itself (that could be even zero), but also on the number of times *c* has been observed in each of the other nodes;
(ii) the probability of *production* (or *exploration*) of a novelty for the entire system by node *h*, i.e. the probability of extracting from urn *h* a color never extracted before from any of the urns in the system, has an increasing dependence not only on the number of novelties produced by *h* itself in the past, but also on the number of novelties produced by each of the other nodes in the past.

In particular, (ii) means that Kauffman’s principle of the adjacent possible is at the “system level”: that is, when node *h* produces a novelty for the system, this fact triggers further potential novelties in all the nodes of the system, not only in node *h* itself. The two different dependencies described above ((i) and (ii)) are tuned by two different matrices (called $\Gamma$ and *W* in the sequel).

Despite the amount of scientific works regarding interacting urns with a finite set of colors (see, for instance^33,34^, and the references therein), in the existing literature we have found only a few papers about a collection of interacting (in the same sense of the present work) urns with infinitely many colors, that is^35–37^. In the model provided in^35^ (see Example 3.8 in that paper), there is a finite collection of Dirichlet processes with random reinforcement. More precisely, in that model we have a random weight $W_{t,h}$ associated to the extraction at time-step *t* from the urn *h* so that, the probability of extracting from urn *h* an old color *c* (here, the term “old” refers to urn *h*, that is a color never extracted before from urn *h*) is proportional to the weight associated to that color, specifically $\sum _{n=1}^t W_{n,h}I_{C_{n,h}=c}/ ( \theta +\sum _{n=1}^t W_{n,h})$. The interaction across the urns is introduced by means of the weights, which could be stochastically dependent: each $W_{n,h}$ may be the same for each urn *h*, or a function of the observed outcomes of the other urns, or a function of some common (observable or latent) variables. It is easy to understand that this model is different from ours: we consider Poisson-Dirichlet processes, not only Dirichlet processes, and, differently from the model in^35^, for us, the notion of “old” or “new” color refers to the entire system, not to each single urn, and Kauffman’s principle of the adjacent possible is at the system level as explained above. Our work and^36^ share the fact that the proposed models are both a collection of urns with triggering with an interacting dynamics that brings the Kauffman’s principle of the adjacent possible from the single agent to the network of agents. Adopting the terminology of^36^, we can say that both interacting mechanisms are based on the construction and the updating of a “social” urn for each network node from which the extractions take place, but the contruction and the updating rules of the social urns are deeply different in the two models. In particular, differently from^36^, we introduce the notion of “new” and “old” at the system level. In^36^ the authors focus on the novelties in each sequence (novelty in *h* = first apperance in *h* of a new item), that they call “discoveries”; while we also study the sequence of the novelties for whole the system produced by each agent. Furthermore, in^36^ the extraction of an “old” item in a certain network node does not affect the other nodes, in our model we also have an interacting reinforcement mechanism for the “old” items: the probability of the extraction of an old item depends on the number of times it has been observed in all the nodes. This allows us to get a specific result on the distribution of the observations in the system among the different items observed. Finally^37^, provides a multi-agent version of the urn with triggering model, which is specific for describing the birth and the evolution of social networks.

While the model we propose is extremely general and may be also employed in other contexts, it has been tested on two real data sets: one taken from the social content aggregation website *Reddit*, collected, elaborated and made freely available on the web by the authors of^38^, and one got from the on-line library *Project Gutenberg*, which is a collection of public domain books. We show that both data sets exhibit empirical behaviours that are in accordance with those predicted by the proven theoretical results.

The sequel of the paper is organized as follows. In “Methods”   we introduce the model and we explain the role played by each model parameter. In “Results”  we illustrate the theoretical results and we show how some real innovation processes can be well described using the proposed model. “Discussion”  is devoted to the discussion of the achieved results and the presentation of possible future developments. Finally, the Supplementary Material collects the analytical proof of all the presented theoretical results.

## Methods

The model we propose essentially consists in a finite system of *interacting* urns with triggering. More precisely, suppose to have *N* urns (that may represent *N* different agents of a system), labeled from 1 to *N*. *At time-step 0, the colors inside each urn are different from those in the other urns*. Let $N_{0,h}>0$ be the number of distinct balls with distinct colors inside the urn *h*. Then, at each time-step $t\ge 1$, one ball is drawn at random from each urn and, for any $h=1,\dots N$, urn *h* is so updated according to the colors extracted from urn *h* itself and from all the other urns $j\ne h$:

- if the color of the ball extracted from urn *h* is “new” (i.e., it appears for the first time in the system), then we replace (inside urn *h*) the extracted ball by ${\widehat{\rho }}_{h,h}>0$ balls of the same color plus $(\nu _{h,h}+1)$, with $\nu _{h,h}\ge 0$, balls of distinct “new” colors (i.e. not already present in the system);
- if the color of the ball extracted from urn *h* is “old” (i.e., it has been already extracted in the system), we add $\rho _{h,h}>0$ balls of the same color into urn *h*;
- for each $j\ne h$, if the color of the ball extracted from urn *j* is “new” (i.e., it appears for the first time in the system), then into urn *h* we add ${\widehat{\rho }}_{j,h}\ge 0$ balls of the same color as the one extracted from urn *j* plus $\nu _{j,h}\ge 0$ balls of distinct “new” colors (i.e. not already present in the system);
- for each $j\ne h$, if the color of the ball extracted from urn *j* is “old” (i.e., it has been already extracted in the system), then into urn *h* we add $\rho _{j,h}\ge 0$ balls of the same color as the one extracted from urn *j*.

As already pointed out, the terms “new” and “old” refer to the entire system, that is a “new” color is a color that has never been extracted from an urn of the system. On the contrary, an “old” color is a color that has already been extracted from at least one urn of the system, but it is possible that it has never been extracted from some other urns in the system.

We assume that the *“new” colors added to a certain urn are always different from those added to the other urns (at the same time-step or in the past)*. By means of this fact, together with the assumption that initially the colors in the urns are different from each other, we cannot have the same new color extracted simultaneously from different urns. In other words, *we cannot have the same novelty produced simultaneously from different agents of the system*. Therefore, for each observed new color (novelty) *c*, there exists a unique urn (agent), say $j^*(c)$, in the system that produced it. However, in a time-step following its first extraction, color *c* could be also extracted from another urn $h\ne j^*(c)$, as a consequence of the interaction among the urns (agents). Indeed, the “contamination” of the color-set of the urn *h* with the colors present in the other urns is possible by means of the interaction terms ${\widehat{\rho }}_{j,h}$ and/or $\rho _{j,h}$ in the above model dynamics.

As in the standard Poisson–Dirichlet model, we assume the *balance condition*

2 $$\begin{aligned} {\widehat{\rho }}_{j,h}+\nu _{j,h}=\rho _{j,h},\quad \text{ i.e. } {\widehat{\rho }}_{j,h}=\rho _{j,h}-\nu _{j,h}, \end{aligned}$$

so that, at each time-step, each urn *j* contributes to increase the number of balls inside urn *h* by $\rho _{j,h}\ge 0$, with $\rho _{h,h}>0$. Therefore, at each time-step, the number of balls added to urn *h* is $\rho _h=\sum _{j=1}^N \rho _{j,h}>0$. Hence, if we denote by $C_{t+1,h}$ the color extracted from urn *h* at time-step $t+1$, we have

$$\begin{aligned} Z^*_{t,h}=P(C_{t+1,h}={\text {``new''}}\,|\, {\text {past}})= \frac{N_{0,h}+\sum _{j=1}^N \nu _{j,h}D^*_{t,j}}{N_{0,h} + \rho _h t}, \end{aligned}$$

where $D^*_{t,j}$ denotes the number, until time-step *t*, of distinct observed colors extracted for their first time from urn *j*, that is the number of distinct novelties for the whole system “produced” by urn (agent) *j* until time-step *t*. Moreover, for each old color *c*, we have

$$\begin{aligned} \begin{aligned} P_{t}(h,c)=P(C_{t+1,h}= c \,|\, \text {past} )&= \frac{\sum _{j\ne j^*(c)}\rho _{j,h}K_t(j,c)+\rho _{j^*(c),h}(K_t(j^*(c),c)-1) +{\widehat{\rho }}_{j^*(c),h}}{N_{0,h} + \rho _h t}\\&=\frac{\sum _{j=1}^N\rho _{j,h}K_t(j,c)+({\widehat{\rho }}_{j^*(c),h} -\rho _{j^*(c),h})}{N_{0,h}+ \rho _h t}\\&=\frac{\sum _{j=1}^N\rho _{j,h}K_t(j,c)-\nu _{j^*(c),h}}{N_{0,h} +\rho _h t}, \end{aligned} \end{aligned}$$

where $K_{t}(j,c)$ denotes the number of times the color *c* has been extracted from urn *j* until time-step *t* and $j^*(c)$ denotes the urn from which the color *c* has been extracted for the first time. (Note that $\rho _{j^*(c),h}=0$ implies $\nu _{j^*(c),h}=0$ by the balance condition.)

Without loss of generality, to ease the notation we adopt a different parametrization by setting

3 $$\begin{aligned} \theta _h=N_{0,h}/\rho _h,\quad \gamma _{j,h}=\nu _{j,h}/\rho _h,\quad \lambda _{j,h}={\widehat{\rho }}_{j,h}/\rho _h\quad \text{ and }\quad w_{j,h}=\rho _{j,h}/\rho _h, \end{aligned}$$

where $\theta _h>0$, $0\le \gamma _{j,h}\le 1$ with $<1$ for $j=h$, $0\le \lambda _{j,h}\le 1$ with $>0$ for $j=h$ and $0\le w_{j,h}\le 1$ with $>0$ for $j=h$. This choice can be read as a normalization of the parameters since, for each $h=1,\dots , N$, we have $\sum _j w_{j,h}=1$ and so, by the balance condition, $0\le \sum _j\gamma _{j,h}<1$ and $0<\sum _j\lambda _{j,h}\le 1$. With the new parametrization, we obtain

4 $$\begin{aligned} Z^*_{t,h}=P(C_{t+1,h}={\text {``new''}}\,|\, {\text {past}})= \frac{\theta _{h}+\sum _{j=1}^N \gamma _{j,h}D^*_{t,j}}{\theta _{h} + t}, \end{aligned}$$

and, for each “old” color *c*,

5 $$\begin{aligned} \begin{aligned} P_{t}(h,c)=P(C_{t+1,h}= c \,|\, {\text {past}} )&= \frac{\sum _{j\ne j^*(c)}w_{j,h}K_t(j,c)+w_{j^*(c),h} (K_t(j^*(c),c)-1)+\lambda _{j^*(c),h}}{\theta _h + t}\\&=\frac{\sum _{j=1}^N w_{j,h}K_t(j,c)-\gamma _{j^*(c),h}}{\theta _h + t}. \end{aligned} \end{aligned}$$

Note that the probability that urn (agent) *h* will produce at time-step $t+1$ a novelty for the entire system has an increasing dependence on the number $D^*_{t,j}$ of novelties produced by the urn (agent) *j* until time-step *t* and the parameter $\gamma _{j,h}$ regulates this dependence. In other words, Kauffman’s principle of the adjacent possible is at the “system level”: that is, for each pair (*j*, *h*) of urns in the system, the parameter $\gamma _{j,h}$ quantifies how much the production of a novelty by urn *j* induces potential novelties in urn *h*. Moreover, on the other hand, the probability that from urn *h* we will extract at time-step $t+1$ an old color *c* has an increasing dependence on the number $K_{t}(j,c)$ of times the color *c* has been drawn from urn *j* until time-step *t* and the parameter $w_{j,h}$ quantifies how much the number $K_t(j,c)$ leads toward a future extraction of a ball of color *c* from urn *h*.

As particular cases, we can see that the case $N=1$ reduces to the classical Poisson-Dirichlet process with parameters $\theta >0$ and $0\le \gamma <1$, and the *case of independence* corresponds to the framework when $w_{j,h}=0$ (and so $\gamma _{j,h}=\lambda _{j,h}=0$) for each $j\ne h$. In the latter case, by the model definition, the colors are not shared by the agents, because each urn has colors different from those inside the other urns. Indeed, for *N* independent Poisson-Dirichlet processes the probability of having colors in common is null.

### Chinese restaurant metaphor

It is also worthwhile to recall that a standard metaphor used to represent the random partition induced by the Poisson-Dirichlet process, that is the random partition of the extracted balls among the observed colors, is the “Chinese restaurant” metaphor: suppose to have a restaurant with infinite tables and, at each time-step $t+1$, a customer enters and sits at a table, with probabilities $Z^*_t$ and $P_{c,t}$ given in (1) as the probability of sitting to an empty table and to an already occupied table, respectively. The random partition induced at time-step *t* is the random allocation of the customers, arrived until time-step *t*, among the occupied tables. The interacting model introduced above can be represented with a similar metaphor. More precisely, suppose to have a restaurant with infinite tables where, at each time-step, *N* customers enter simultaneously. Each customer belongs to a specific category $h=1,\dots , N$. Then, at time-step $t+1$, the probability that the customer belonging to category *h* sits to an empty table is $Z^*_{t,h}$ defined in (4) and the probability that she sits to an already occupied table is $P_t(h,c)$ defined in (5). We cannot have customers belonging to different categories that occupy simultaneously the same empty table. However, the sharing of a table by multiple categories is possible, after the first occupation of the table, because of the presence of the interaction terms $\lambda _{j,h}$ and $w_{j,h}$ in (5). The probability $Z^*_{t,h}$ results increasing not only with the number of distinct tables occupied by customers of category *h* until time-step *t*, but also with the numbers of distinct tables occupied by customers of each other category $j\ne h$. The parameters $\gamma _{j,h}$ rule these dependencies. Similarly, the probability $P_t(h,c)$ has naturally an increasing dependence on the number of customers already seated at that table, but each of these customers has a different weight, i.e. $w_{j,h}$, according to her category: indeed, the parameter $w_{j,h}$ regulates how much the number of customers of category *j* sitting to a table drives a customer of category *h* to choose that table. For the sake of clarity, we have synthetize in Table 1 how the quantities and the events involved in the proposed model can be interpreted through both the urn metaphor or the Chinese restaurant metaphor.

**Table 1:** Correspondence table between the model, the urn metaphor and the Chinese restaurant metaphor.

	Urn metaphor	Chinese restaurant metaphor
Agents	Urns	Categories
Agent’s action	Extracted ball	Customer entering the restaurant
Item adopted	Color of the extracted ball	Table chosen by the customer
Production (or exploration) of a novelty	Extraction of a color never extracted before from any urn of the system	Occupation of an empy table
Exploitation of an old item	Extraction of a color already extracted before from some urn in the system	Choice of an already occupied table
$D^*_{t,h}=$ number, until time-step *t*, of distinct novelties for the whole system produced by agent *h*	Number, until time-step *t*, of distinct colors observed in the whole system and extracted for their first time from urn *h*	number, until time-step *t*, of distinct tables occupied for their first time by a customer belonging to category *h*
$D_{t,h}=$ number, until time-step *t*, of distinct items adopted by agent *h*	Number, until time-step *t*, of distinct colors extracted from urn *h*	Number, until time-step *t*, of distinct tables occupied by at least one customer belonging to category *h*
$K_t(h,c)=$ number, until time-step *t*, of times agent *h* has adopted item *c*	Number, until time-step *t*, of times color *c* has been extracted from urn *h*	Number, until time-step *t*, of customers belonging to category *h* and sitting at table *c*

### Matrix notation

In order to present the theoretical results, we set $\Gamma$, *W*, $\Lambda$ equal to the non-negative $N\times N$ square matrices with elements $\gamma _{j,h}$, $w_{j,h}$ and $\lambda _{j,h}$, respectively. We recall that, by the balance condition (2) and the reparametrization (3), we have

$$\begin{aligned} W=\Gamma +\Lambda ,\qquad {\textbf{0}}^\top \le {{{\textbf{1}}}}^\top \Gamma <{{\textbf{1}}}^\top \qquad \text{ and }\qquad {{{\textbf{1}}}}^\top W={{{\textbf{1}}}}^\top , \end{aligned}$$

where ${{{\textbf{1}}}}$ and ${{{\textbf{0}}}}$ denote the vectors with all the components equal to 1 and 0, respectively. As observed above, the matrix $\Gamma$ rules the production of potential novelties and, in particular, its elements out of the diagonal regulate the interaction among the agents with respect to this issue; while, the matrix *W* rules the interaction among the agents with respect to the choice of an old item.

## Results

In this section we will present first the theoretical results and then the empirical results related to two real data sets. The proofs of the first ones are collected in the Supplementary Materials, that may be found together with the online version at^39^.

### Theoretical results

The first result states that, if $\Gamma$ is irreducible, that is the graph with the agents as nodes and with $\Gamma$ as the adjacency matrix is strongly connected, then $D^*_{t,h}\propto t^{\gamma ^*}$ a.s. for all $h=1,\dots ,N$, that is all the $D^*_{t,h}$ grow with the same Heaps’ exponent $\gamma ^*\in (0,1)$. This means that, at the steady state, all the agents of the network produce innovations for the system at the same rate. In addition, the ratio $D^*_{t,h}/D^*_{t,j}$ provides a strongly consistent estimator of the ratio $u_h/u_j$ of the relative centrality scores (with respect to $\Gamma ^\top$) of the two nodes *h* and *j*. More precisely, we have

#### Theorem 3.1

*Suppose that the matrix* $\Gamma$ *is irreducible. Denote by* $\gamma ^*\in (0,1)$ *the Perron–Frobenius eigenvalue of* $\Gamma$*, by* ${\textbf{v}}$ *the corresponding right eigenvector with strictly positive entries and such that* ${\textbf{v}}^\top {{{\textbf{1}}}}=1$ *and, finally, denote by* ${\textbf{u}}$ *the corresponding left eigenvector with strictly positive entries and* ${\textbf{v}}^\top {\textbf{u}}=1$. *Then, for each* $h=1,\dots , N$*, we have*

$$\begin{aligned} t^{-\gamma ^*}D^*_{t,h}{\mathop {\longrightarrow }\limits ^{a.s.}}D^{**}_{\infty ,h}, \end{aligned}$$

*where* $$D^{**}_{\infty ,h}$$ *is a finite strictly positive random variable. Moreover, for each pair of indexes* $$h,j=1,\dots , N$$*, we have*

$$\begin{aligned} \frac{ D^{*}_{t,h} }{ D^{*}_{t,j} }{\mathop {\longrightarrow }\limits ^{a.s.}}\frac{u_h}{u_j}. \end{aligned}$$

As a consequence, since the number $D_t^*$ of distinct items observed in the entire system until time-step *t* coincides by model definition with $\sum _{h=1}^N D^*_{t,h}$, we also have that this number grows as $t^{\gamma ^*}$, i.e.

$$\begin{aligned} t^{-\gamma ^*}D^*_t{\mathop {\longrightarrow }\limits ^{a.s.}}D_\infty ^{**}=\sum _{h=1}^N D^{**}_{\infty ,h}. \end{aligned}$$

Furthermore, if we denote by $(D_{t,h})$ the *discovery* process^36^ for agent *h*, that is if we denote by $D_{t,h}$ the number of distinct items adopted by agent *h*, then we have $D^*_{t,h}\le D_{t,h}\le D^*_t$ and so we get

$$\begin{aligned} D_{t,h}=O(t^{\gamma ^*})\qquad \text{ and }\qquad 1/D_{t,h}=O(t^{-\gamma ^*}), \end{aligned}$$

which, in particular, imply that, when the quantities $D_{t,h}$ have an asymptotic power law behavior, then they necessarily have the same Heaps’ exponents, equal to $\gamma ^*$. In addition, we obtain

$$\begin{aligned} \frac{u_h}{\sum _{h=1}^N u_h}\le \liminf _{t}\frac{D_{t,h}}{D_{t,j}}\le \limsup _t\frac{D_{t,h}}{D_{t,j}} \le \frac{\sum _{h=1}^N u_h}{u_j}. \end{aligned}$$

The second result of the present work affirms that if *W* is irreducible, that is the graph with the agents as nodes and *W* as the adjacency matrix is strongly connected, then, for each observed item *c*, the number of times item *c* has been adopted by agent *h* grows linearly. Moreover, at the steady state, the times item *c* has been adopted in the whole system are uniformly distributed among the agents. This concept can be reformulated more clearly using the metaphor of the Chinese restaurant: the limit composition of each table *c* is the uniform one (with respect to the categories). More precisely, we have

#### Theorem 3.2

*Suppose that the matrix* *W* *is irreducible. Then, for each* $h=1,\dots ,N$*, we have*

$$\begin{aligned} \frac{1}{t}K_t(h,c){\mathop {\longrightarrow }\limits ^{a.s.}}K_{\infty }(c) \end{aligned}$$

*for each observed color* *c* *in the system, where* $K_{\infty }(c)$ *is a suitable random variable that takes values in* (0, 1] *and does not depend on* *h*. *As a consequence, for each* $h=1,\dots ,N$, *we also have that*

$$\begin{aligned} \frac{K_t(h,c)}{\sum _{j=1}^N K_t(j,c)}{\mathop {\longrightarrow }\limits ^{a.s.}}\frac{1}{N}. \end{aligned}$$

### Empirical results

In this subsection we show that the behaviors predicted by the previous theoretical results match with the ones we actually observe in two different real data sets: one taken from the social content aggregation website *Reddit*, collected, elaborated and made freely available on the web by the authors of^38^ at https://github.com/corradomonti/demographic-homophily, and one got from the on-line library *Project Gutenberg* at https://www.gutenberg.org/. In order to illustrate these examples, we adopt the metaphor of the Chinese restaurant and so, for each of them, we identify the customers’ categories and the tables we are looking at. In both examples, we consider $N=2$ categories with their sequences of customers who select the tables. See Table 2 for a guide on how to interpret the quantities and the events of interest in the considered data sets in terms of the Chinese restaurant metaphor.

We analyze the processes $(D^*_{t,h})$ and $(D_{t,h})$, with $h=1,\,2$, and the composition of the tables, constructed starting from the real data, in order to verify if they exhibit a behavior along time in agreement with the theoretical results of the previous section. Specifically, we point out:

1. the power law behavior of the processes $(D^*_{t,h})$ and $(D_{t,h})$, with $h=1,2;$
2. the fact that the above processes increases with the same Heaps’ exponent (the constant $\gamma ^*$ in Theorem 3.1);
3. the convergence of the ratio $D^*_{t,1}/D^*_{t,2}$, or equivalently of the difference $\log _{10}(D^*_{t,1})-\log _{10}(D^*_{t,2})$, as $t\rightarrow +\infty$;
4. the convergence of the composition of the tables toward the uniform one (as stated in Theorem 3.2).

For points (1) and (2), we follow the standard method in literature: we provide the $\log _{10}-\log _{10}$ plot of the considered processes and the estimate of the common slopes of the corresponding lines by a least square interpolation. The goodness of fit of the provided lines with the same slope is supported by the extremely high value of the $R^2$ index. Regarding point (3), we plot the observed sequence $\log _{10}(D^*_{t,1})-\log _{10}(D^*_{t,2})$ along time, in order to highlight how its fluctuations decrease along time and how it asymptotically stabilizes. The limit of this process is estimated as the difference between the intercepts of the two lines obtained for $D^*_{t,1}$ and $D^*_{t,2}$ in the $\log _{10}-\log _{10}$ plot. This value, denoted as ${\widehat{u}}$, represents an estimation of the difference $\log _{10}(u_1/u_2)=\log _{10}(u_1)-\log _{10}(u_2)$, where $r=u_1/u_2$ is the limit quantity in the second part of Theorem 3.1, which is also the ratio of the two centrality scores with respect to $\Gamma ^\top$ of the two categories. Finally, for point (4), we plot the quantiles of the distribution of the proportion $\frac{K_t(1,c)}{K_t(1,c) + K_t(2,c)}$, from the least populated table *c* to the most populated one, in order to appreciate their convergence toward 1/2.

**Table 2:** Correspondence table for applications.

Chinese restaurant metaphor	Reddit data set	Gutenberg data set
Categories	Sentiment categories (positive/negative)	Literary genres (western/history)
Customer entering the restaurant	Comment	Word
Chosen table	Author of the commented news	Word
Occupation of an empty table	First comment to a news	First appearance of a word
Choice of an already occupied table	Comment to a news whose author has already received comments to other her news	Usage of a word already used before in some of the literary genres
$D^*_{t,h}=$ number, until time-step *t*, of distinct tables occupied for their *first* time by a customer belonging to category *h*	Number, until time-step t, of distinct authors whose *first* received comment belongs to sentiment category *h*	Number, until time-step *t*, of distinct words whose *first* apperance has been in literary genre *h*
$D_{t,h}=$ number, until time-step *t*, of distinct tables occupied by *at least* one customer belonging to category *h*	Number, until time-step *t*, of distinct authors who have received *at least* one comment belonging to sentiment category *h*	Number, until time-step *t*, of distinct words that have been used in literary genre *h*
$K_t(h,c)=$ number, until time-step *t*, of customers belonging to category *h* and sitting at table *c*	Number, until time-step *t* of comments received by author *c* belonging to sentiment category *h*	Number, until time step *t*, of times the word *c* has been used in literary genre *h*

#### Reddit data set

This data set consists of a collection of news, and comments associated to each news, for the period 2016–2020, downloaded from the *r/news* community on the website *Reddit* at https://www.reddit.com/r/news, which is devoted to the discussion of news articles about events in the United States and the rest of the world. Each news is associated with the author who posted it. Moreover, the data set contains the specific topic the news belongs to (we refer to^38^ for details about the topic classification) and, to each comment is also assigned a measurement of the sentiment, expressed as a real value in $(-1,1)$. It corresponds to the “compound” score given by the VADER (Valence Aware Dictionary and sEntiment Reasoner) Sentiment Analysis^40^, which is a lexicon and rule-based sentiment analysis tool, specifically thought for sentiments expressed in social media.

Here we consider only the comments to news belonging to the topic “Politics”. Moreover, we categorize the sentiment variable, following^41^: precisely, we define it as “positive” if the provided sentiment value was larger than $+0.35$ and “negative” if the provided sentiment value was lower than $-0.35$. Any comment with an original sentiment value that lies within $-0.35$ and $+0.35$ has been removed. Summing up, we consider all the comments to the commented news regarding the topic “Politics”, with a sentiment value larger than $+0.35$ (positive) or lower than $-0.35$ (negative). This provides us a total of 3, 016, 990 comments in the negative sentiment category and 2, 602, 173 comments in the positive sentiment category.

We are interested in the sequence of authors who receive at least one comment with negative sentiment for the news they post and in the analogous sequence related to comments with positive sentiment. As explained above, we illustrate that these two sequences exhibit the asymptotic behaviors predicted by the proposed model. For this purpose, we firstly identify the main quantities related to the Chinese restaurant version of the model: each sentiment category is a customer category (category 1 = negative sentiment and category 2 = positive sentiment) and the authors represent the tables. Therefore, when at time-step *t* a news receives a comment with a specific sentiment, then the author who posted such a news is “new” or “old” for that specific sentiment category if, respectively, she has or has not already received a comment within that sentiment category. Analogously, the author will be “new” or “old” for the entire system (the whole collection of comments) if, respectively, she has or has not already received any comment. In order to obtain two sequences of comments of the same length, as required by the model, we have randomly removed some comments from the negative sentiment category, i.e. the one containing more comments. In addition, we verified successfully that an author is not commented for the first time simultaneously with two comments of different sentiment.

**Figure 1:**
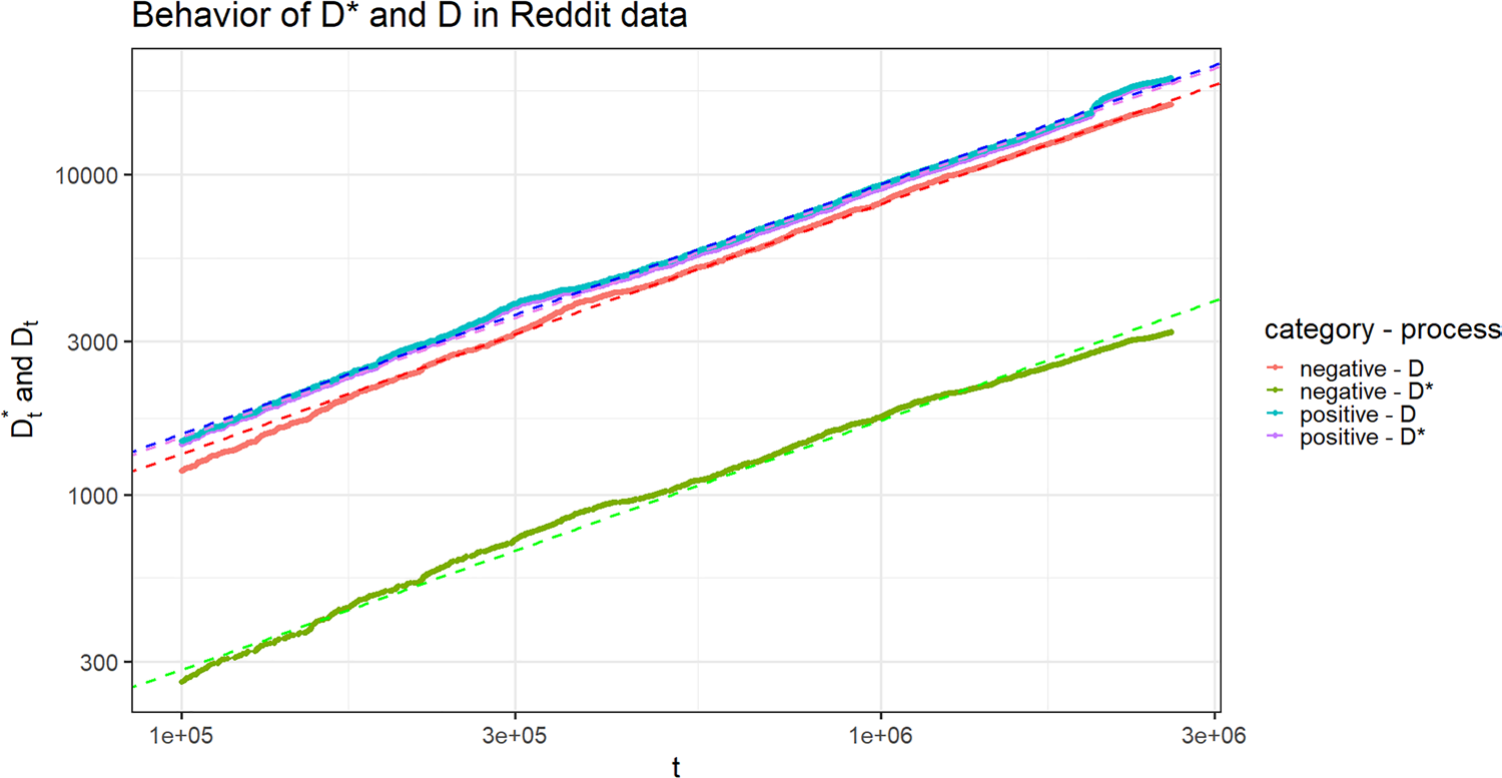
Reddit data set. Linear behavior of $(D^{*}_{t,h})$ and $(D_{t,h})$ along time, for $h=1,2$, in $\log _{10}-\log _{10}$ scale. The dashed lines are obtained by a least square interpolation. The goodness of fit $R^2$ index is 0.9984. The estimated common slop is ${\widehat{\gamma }}^*=0.781$.

**Figure 2:**
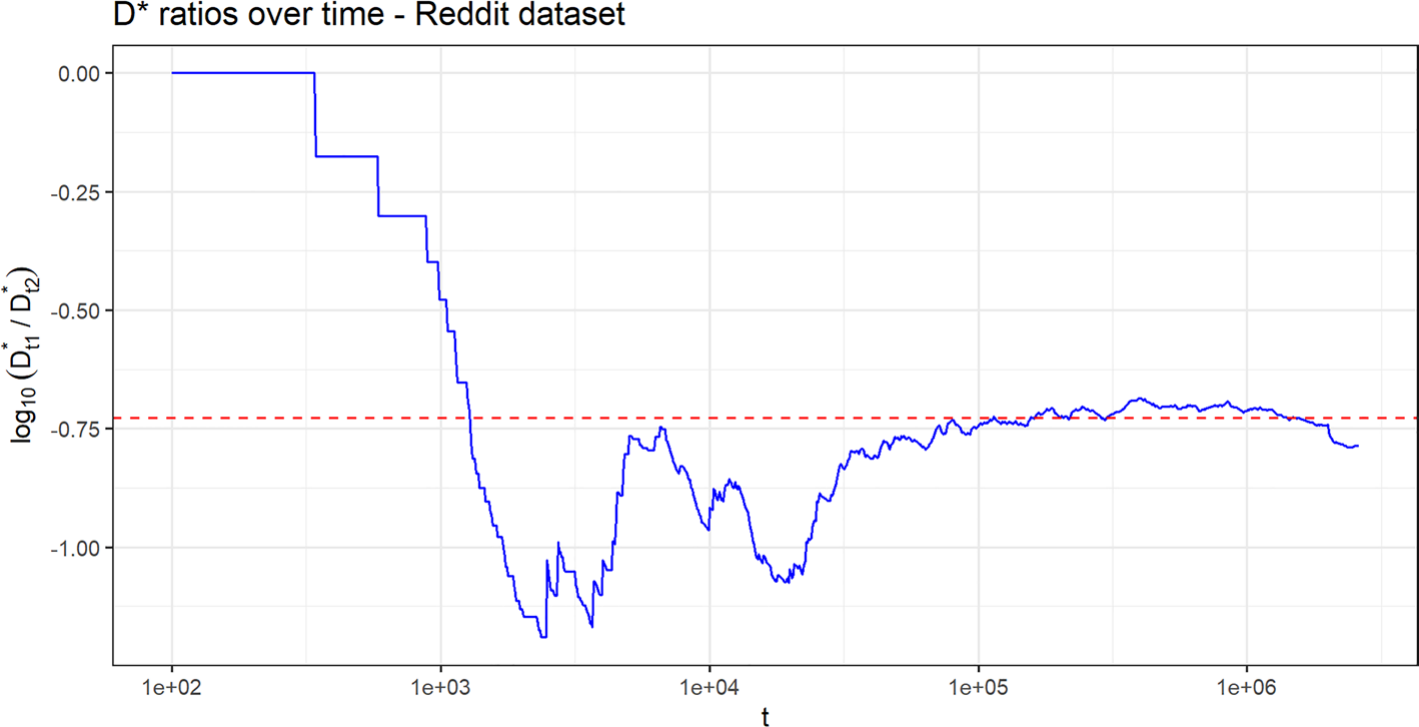
Reddit data set. Plot of the process $\log _{10}(D^*_{t,1})-\log _{10}(D^*_{t,2})$ along time. The horizontal dashed red line represents the estimated limit value ${\widehat{u}}=-0.727$.

For each possible sentiment category $h=1,\,2$, the observed quantity $D^*_{t,h}$ (i.e. the number, until time-step *t*, of distinct authors whose *first* received comment belongs to sentiment category *h*) shows a power law growth along time. We can observe the same behavior for $D_{t,h}$ (i.e. the number, until time-step *t*, of distinct authors who have received *at least one* comment belonging to sentiment category *h*). Figure 1 provides the asymptotic behavior of these processes in $\log _{10}-\log _{10}$ scale, where we can also appreciate how the lines exhibit the same slope, which indicates that the processes have the same Heaps’ exponent. This is exactly in accordance with the first result of Theorem 3.1. The estimated value of the Heaps’ exponent, estimated as the common slope of the lines in the $\log _{10}-\log _{10}$ plot is ${\widehat{\gamma }}^*=0.781$. Figure 2 shows the convergence of the process $\log _{10}(D^*_{t,1})-\log _{10}(D^*_{t,2})$ toward the estimated limit value ${\widehat{u}}=-0.727$, computed as the difference between the intercepts of the two regression lines for the two processes $(D^*_{t,h})$ in Fig. 1. This value is an estimation of the quantity $u=\log _{10}(r)$, where $r=u_1/u_2=10^u$ is the limit in the second result of Theorem 3.1.

**Figure 3:**
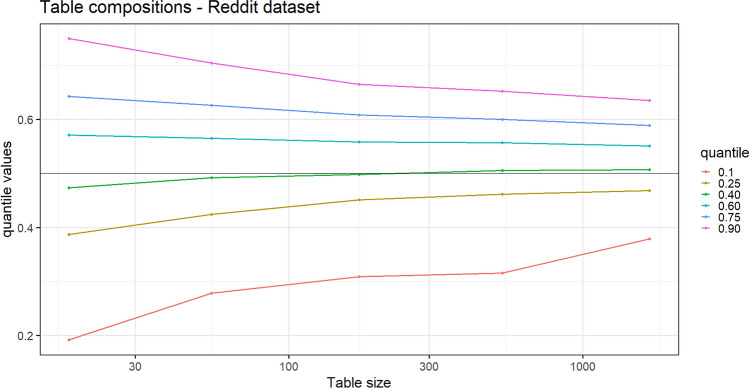
Reddit data set. Real data: quantiles of the distribution of the proportion of comments with negative sentiment received by the authors along their number of received comments, form the least commented to the most commented.

Regarding the table composition, we provide Fig. 3 with the proportion of comments with negative sentiment received by an author over the total number of received comments. More precisely, we plot the quantiles of the empirical distribution of this proportion, from the least commented author to the most commented one. In order to construct the quantiles, we have listed the authors (tables) from the least commented to the most commented, removing those commented less than ten times (tables with less than 10 customers), then we have grouped these authors taking intervals of equal length (0.5 in $\log _{10}$ scale). Finally, within each group, we have computed the quantiles of the empirical distribution of the proportion of the comments with negative sentiment the authors have received with respect to the total number of received comments. We can appreciate how these quantiles get closer to 1/2 (the uniform composition) as the number of received comments increases. This is in accordance with Theorem 3.2.

#### Gutenberg data set

We downloaded this data set from the on-line library *Project Gutenberg*. It consists of a collection of over 70, 000 free ebooks. After selecting only those written in English and classifying them in different topics, we decided to focus on two particular literary genres: “Western” and “History”. For each of them, we have considered all the words contained in seven books, for a total of 480, 460 words for “Western” and 476, 948 words for “History” (after a slight pre-processing: e.g. removal of punctuation, spaces, numbers and words with 1 or 2 characters and acquisition of the stem of the words by means of Dr. Martin Porter’s stemming algorithm^42^).

We are interested in the two sequences of words for the two different literary genres and, as explained at the beginning of this subsection, we would like to check that these two sequences exhibit the asymptotic behaviors predicted by the proposed model. In order to do so, we firstly identify the main quantities related to the Chinese restaurant version of the model: each literary genre is a customer category (category 1 = “Western” and category 2 = “History”) and the words represent the tables. Therefore, each word will be “new” or “old” for a specific literary genre if, respectively, it has or has not already been used within that genre. Analogously, each word will be “new” or “old” for the entire system if, respectively, it has or has not already been used within any considered book. In order to obtain two sequences of words of the same length, as required by the model, we have randomly removed some words from the category “Western”, i.e. the one containing more words. In addition, we verified successfully that a new word does not appear for the first time simultaneously in both genres.

**Figure 4:**
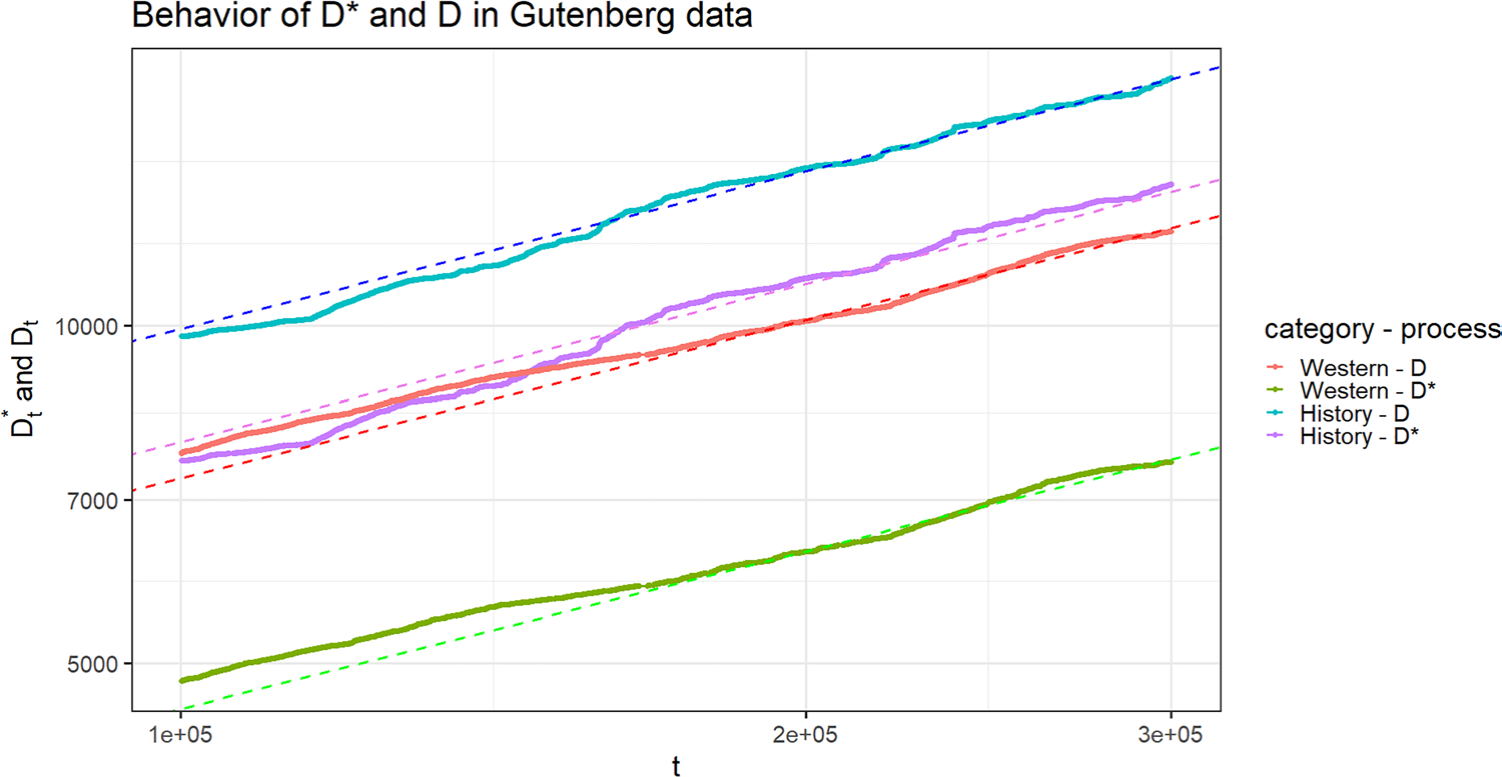
Gutenberg data set. Linear behavior of $(D^{*}_{t,h})$ and $(D_{t,h})$ along time, for $h=1,2$, in $\log _{10}-\log _{10}$ scale. The dashed lines are obtained by a least square interpolation. The goodness of fit $R^2$ index is 0.9937. The estimated common slope is ${\widehat{\gamma }}^*=0.466$.

**Figure 5:**
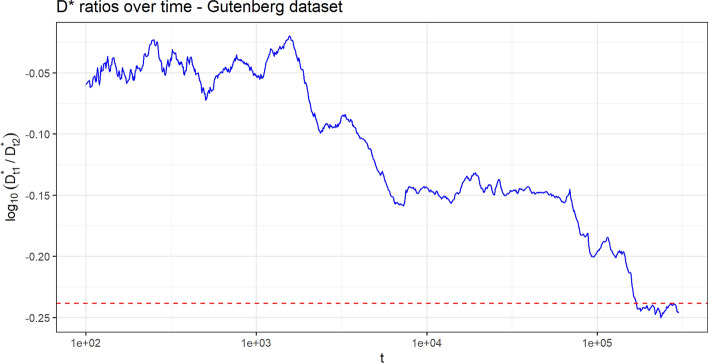
Gutenberg data set. Plot of the process $\log _{10}(D^*_{t,1})-\log _{10}(D^*_{t,2})$ along time. The horizontal dashed red line represents the estimated limit value ${\widehat{u}}=-0.238$.

For each literary genre $h=1,\,2$, the observed quantity $D^*_{t,h}$ (i.e. the number, until time-step *t*, of distinct words whose *first* appearance has been in literary genre *h*) shows a power law growth along time. The same behavior is shown by $D_{t,h}$ (i.e. the number, until-time-step *t*, of distinct words used in literary genre *h*). Figure 4 provides the asymptotic behavior of these processes in $\log _{10}-\log _{10}$ scale, where we can also appreciate how the lines exhibit the same slope, which indicates that the processes have the same Heaps’ exponent. This is exactly in accordance with the first result of Theorem 3.1. The estimated value of the common Heaps’ exponent, estimated as the common slope of the lines in the $\log _{10}-\log _{10}$ plot is ${\widehat{\gamma }}^*=0.466$. Figure 5 shows the convergence of the process $\log _{10}(D^*_{t,1})-\log _{10}(D^*_{t,2})$ toward the estimated limit value ${\widehat{u}}=-0.238$, computed as the difference between the intercepts of the two lines for the two processes $(D^*_{t,h})$ in Fig. 4. This value is an estimation of the quantity $u=\log _{10}(r)$, where $r=u_1/u_2=10^u$ is the limit in the second result of Theorem 3.1. With respect to the Reddit data set, we can observe that here the convergence is slower.

**Figure 6:**
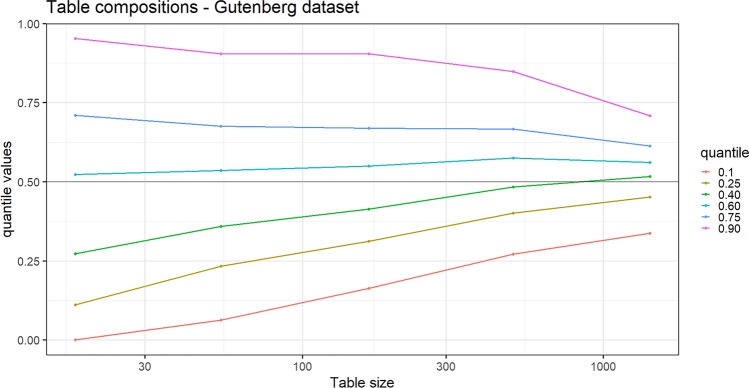
Gutenberg data set. Real data: quantiles of the distribution of the proportion of times the words have been used in the topic “western” along their frequency in the system, form the least frequent to the most frequent.

Regarding the table composition, we provide Fig. 6 with the proportion of times a word has been used in the topic “Western” over the total number of times it has been used in the entire system. As in the previous application, we plot the quantiles of the empirical distribution of this proportion along the frequency of the words in the system, from the least frequent to the most frequent. In order to construct the quantiles, we have listed the words (tables) from the least frequent to the most frequent, removing those appeared less than 10 times (tables with less than 10 customers), then we have grouped these words taking intervals of equal length (0.5 in $\log _{10}$ scale). Finally, within each group, we have computed the quantiles of the empirical distribution of the proportion of times the words have been used in the topic “Western” with respect to the total number of times it has been used in the entire system. We can appreciate how these quantiles get closer to 1/2 (the uniform composition) as the frequency of the word increases. This is in accordance with Theorem 3.2.

## Discussion

In this work we have introduced a general model in order to analyze a system of *N* *interacting* innovation processes. The interaction among the processes is ruled by two matrices $\Gamma$ and *W*. The first one regulates the production of potential novelties, while the second one tunes the interaction with respect to the choice of an old item. When matrix $\Gamma$ is irreducible, we have proven that the numbers $D^*_{t,h}$, with $h=1,\dots , N$, of distinct novelties for the entire system produced by agent *h* until time-step *t* have and asymptotic power law behavior with a common Heaps’ exponent $0<\gamma ^*<1$. Moreover, we have proven that the ratio $D^*_{t,h}/D^*_{t,j}$ converges almost surely toward the ratio $u_h/u_j$ of the relative centrality scores of *h* and *j*. Finally, when the matrix *W* is irreducible, we have proven that, for each observed item *c*, the number of times item *c* has been adopted by agent *h* (i.e. the number of customers of category *h* sitting at table *c*) grows linearly and the proportions of times it has been adopted by agent *h* over the number of times it has been adopted in general in the system converges almost surely to 1/*N* (i.e. the asymptotic composition of table *c*, with respect to the different *N* categories, is the uniform one).

In order to highlight the potentialities of the proposed model and of the proven related results in the study of the interaction among innovation processes, we have illustrated that the behaviors predicted by the provided theoretical results match with the ones we observe in two real data sets. One interesting research line that we have in mind for the future is to study the speed of convergence for the limits given in the shown theoretical results, in order to develop statistical instruments for an accurate inference on the two interaction matrices, $\Gamma$ and *W*, from the real data. Regarding this issue, it is important to note that the value $\gamma ^*$ and the vector ${\textbf{u}}=(u_h)_{h=1,\dots ,N}$ do not uniquely determine the matrix $\Gamma$. In other terms, given the estimates of $\gamma ^*$ and of ${\textbf{u}}$, there exist infinite matrices $\Gamma$ that could have generated that estimated values. This is a tough point to deal with and further theoretical results are needed if we want to detect the model parameters from the data. In the Supplementary Material, we present an idea for a first estimation of the interaction matrices in the case $N=2$: after the estimation of $\gamma ^*$ and $\log _{10}(r)=\log _{10}(u_1/u_2)$ as the common slope and the difference of the intercepts, respectively, of the lines related to the observed processes $(D^*_{t,h})$, $h=1,\,2$, plotted in $\log _{10}-\log _{10}$ scale, we can consider parametric families of matrices $\Gamma$ and *W* compatible with these estimated values and we can perform a Maximum Likelihood Estimation (MLE) in order to detect the remaining parameters that better fit the data. However, for having a robust MLE procedure, we need to reduce the number of parameters by imposing some restrictions on them, for instance the symmetry of the matrices. We have tested this procedure on some simulations and the results are collected in the Supplementary Material.

Regarding the model assumptions, we point out that the balance condition (2) forces to have Heaps’ exponents strictly smaller than 1. Since eliminating this condition in the case of a single process ($N=1$) makes an exponent equal to 1 possible^28–31^, it is plausible that it would be the same also for $N\ge 2$. Therefore, a second research line for the future is to investigate the proposed model without assuming the balance condition. Moreover, the balance condition forces $w_{h,j}$ (the parameter governing the interaction in the selection of an old item) to be large whenever $\gamma _{h,j}$ (the parameter tuning the interaction with respect to the production of potential novelties) is large and, vice versa, $\gamma _{h,j}$ is necessarily small whenever $w_{h,j}$ is small. On the contrary, the proposed model without the restriction of the balance condition may include cases where $\gamma _{h,j}$ is large, but $w_{h,j}$ is small.

Another model assumption that could be removed is the simultaneity in the extractions from all the urns of the system (i.e. in the arrivals of the customers for all the categories). Indeed, this condition forces to have the same number of observations for each process of the system. This variant of the model could be obtained by inserting a selection mechanism for the urn from which the extraction at a certain time-step will be performed (i.e. for the category of the customer who will enter the restaurant at a certain time-step). This selection could be driven by a reinforcement mechanism on the number of times an urn (category) has been selected.

Finally, regarding the assumption of irreducibility in the theoretical results, we underline that when the matrices are not irreducible, it is possible to decompose them in irreducible sub-matrices such that the union of the spectra of the sub-matrices coincides with the spectrum of the original matrix. Then, a deeper analysis starting from the present theory is needed in the same spirit of^33,34,43^. See also the Supplementary Material for an heuristic argument in order to deduce the rate at which each $D^*_{t,h}$ grows in the case of a general (i.e. not necessarily irreducible) matrix $\Gamma$. The proofs of the theoretical results and other details are available online at^39^. Auxiliary empirical analyses are available online at^44^.

## Supplementary Information

Supplementary Information 1.

Supplementary Information 2.

## Data Availability

The data set Reddit is freely available at https://github.com/corradomonti/demographic-homophily, and has been provided by the authors of^38^. The data set from Gutenberg contains books freely used in the United States because they are not protected by U.S. copyright law. All data generated during this study are included in this published article and its supplementary information files.
