# Supplementary material for: Interacting innovation processes
Source: Sci Rep. 2023 Oct 11;13:17187. doi: 10.1038/s41598-023-43967-1 (PMC10567777; doi:10.1038/s41598-023-43967-1)
Supplement: Supplementary file 1 — Supplementary Information 1. [file 41598_2023_43967_MOESM1_ESM.pdf]

Supplementary material for Interacting Innovation processes

Giacomo Aletti[1], Irene Crimaldi[2], and Andrea Ghiglietti[3*]

[1]ADAMSS Center, Università degli Studi di Milano, Milan, Italy
[2]IMT School for Advanced Studies Lucca, Lucca, Italy
[3]Università degli Studi di Milano-Bicocca, Milan, Italy
[*]Corresponding author: andrea.ghiglietti@unimib.it

ABSTRACT

In the present supplementary file we provide the datailed proofs of the theoretical results given in the main article. The martingale theory is the key issue to prove these results.

S1 Analytical proofs

Denote by $X^*_{t,h}$ the random variable that takes value 1 when the ball extracted from urn h at time-step t has a new (for all the system) color and is equal to 0 otherwise. Then $Z^*_{t,h}$ defined in (4) coincides with $P(X^*_{t+1,h}=1\,|\,\text{past}) = E[X^*_{t+1,h}\,|\,\text{past}]$ and $D^*_{t,h}$ can be written as $\sum_{n=1}^{t} X^*_{n,h}$. Since we have

$$Z^*_{t,h} = \frac{\theta_h + \sum_{n=1}^{t}\sum_{j=1}^{N}\gamma_{j,h}X^*_{n,j}}{\theta_h + t}\,,$$

we obtain the following dynamics for $Z^*_{t,h}$:

$$Z^*_{0,h}=1, \qquad Z^*_{t+1,h} = (1-r_{t,h})Z^*_{t,h} + r_{t,h}\sum_{j=1}^{N}\gamma_{j,h}X^*_{t+1,j} \quad \text{for } t\geq 0\,,$$

where $r_{t,h} = 1/(\theta_h+t+1) = 1/(t+1) + O_h(1/t^2)$. The corresponding vectorial dynamics for $\mathbf{Z}^*_t = (Z^*_{t,1},\dots,Z^*_{t,N})^\top$ is

$$\begin{aligned}
\mathbf{Z}^*_0 &= \mathbf{1}\\
\mathbf{Z}^*_{t+1} &= \left(1-\frac{1}{t+1}\right)\mathbf{Z}^*_t + \frac{1}{t+1}\Gamma^\top\mathbf{X}^*_{t+1} + O(1/t^2)\\
&= \mathbf{Z}^*_t - \frac{1}{t+1}(I-\Gamma^\top)\mathbf{Z}^*_t + \frac{1}{t+1}\Gamma^\top\Delta\mathbf{M}^*_{t+1} + O(1/t^2) \quad \text{for } t\geq 0,
\end{aligned} \tag{S:1}$$

where $\Delta\mathbf{M}^*_{t+1} = \mathbf{X}^*_{t+1} - \mathbf{Z}^*_t$ and $O(1/t^2) = (O_1(1/t^2),\dots,O_N(1/t^2))^\top$.

We prove the following key result:

Theorem S1.1. *Under the same assumptions and notation of Theorem 3.1, we have*

$$t^{1-\gamma^*}\mathbf{Z}^*_t \xrightarrow{a.s.} \widetilde{Z}^{**}_\infty\mathbf{u}\,,$$

where $\widetilde{Z}^{**}_\infty$ is an integrable strictly positive random variable.

Proof. We firstly want to decompose the vectorial process $\mathbf{Z}^*_t$ based on the Jordan representation of the matrix Γ. Specifically, for any $\gamma\in Sp(\Gamma^\top)\setminus\gamma^*$, we can denote as J_γ the Jordan block and with U_γ and V_γ the matrices whose columns are, respectively, the left and right (possibly generalized) eigenvectors of Γ associated to the eigenvalue γ, i.e.

$$\Gamma V_\gamma = V_\gamma J_\gamma \qquad \text{and} \qquad U_\gamma^\top\Gamma = J_\gamma U_\gamma^\top\,.$$

Then, we can consider the decomposition

$$\mathbf{Z}_t^* = \widetilde{Z}_t^* \mathbf{u} + \sum_{\gamma \in Sp(\Gamma^\top)\setminus\gamma^*} \mathbf{Z}_{\gamma,t}^*,$$

where $\widetilde{Z}_t^* = \mathbf{v}^\top \mathbf{Z}_t^*$ and $\mathbf{Z}_{\gamma,t}^* = U_\gamma V_\gamma^\top \mathbf{Z}_t^*$. Secondly, we set

$$\zeta_0 = 1, \qquad \zeta_t = C / \prod_{k=1}^{t} \left[1 - \frac{(1-\gamma^*)}{k}\right] \sim t^{1-\gamma^*} \uparrow +\infty,$$

where C is a suitable constant > 0, and

$$\mathbf{Z}_t^{**} = \zeta_t \mathbf{Z}_t^*, \quad \widetilde{Z}_t^{**} = \zeta_t \widetilde{Z}_t^* \quad \text{and} \quad \mathbf{Z}_{\gamma,t}^{**} = \zeta_t \mathbf{Z}_{\gamma,t}^*$$

(note that $\widetilde{Z}_t^{**}$ is non-negative but not bounded by 1 as $\widetilde{Z}_t^*$) so that we have

$$\mathbf{Z}_t^{**} = \widetilde{Z}_t^{**} \mathbf{u} + \sum_{\gamma \in Sp(\Gamma^\top)\setminus\gamma^*} \mathbf{Z}_{\gamma,t}^{**}.$$

In the following steps, we are going to show that $\widetilde{Z}_t^{**}$ converges almost surely and in mean to an integrable random variable $\widetilde{Z}_\infty^{**}$ such that $P(\widetilde{Z}_\infty^{**} > 0) = 1$ and that each $\mathbf{Z}_{\gamma,t}^{**}$ converges almost surely to zero. In particular, this last task will be done separately for the eigenvalues with $|\gamma| < \gamma^*$ and with $|\gamma| = \gamma^*$. Remember that the assumption that Γ (or, equivalently, $\Gamma^\top$) is irreducible ensures that γ^* is real, simple and $|\gamma| \leq \gamma^*$ for any $\gamma \in Sp(\Gamma^\top)$. In the sequel of the proof, the symbol $\mathscr{F}_t$ denotes the past until time-step t.

Study of $\widetilde{Z}_t^{**}$. By multiplying equation (S:1) by $\mathbf{v}^\top$ we obtain

$$\widetilde{Z}_0^* = 1, \qquad \widetilde{Z}_{t+1}^* = \left[1 - \frac{1}{t+1}(1-\gamma^*)\right] \widetilde{Z}_t^* + \frac{1}{t+1}\gamma^* \Delta \widetilde{M}_{t+1}^* + \widetilde{O}\left(\frac{1}{t^2}\right).$$

Then, multiplying everything by ζ_{t+1} and using the relation $\zeta_{t+1} = \zeta_t[1-(1-\gamma^*)/(t+1)]^{-1}$ we get the following dynamics for $\widetilde{Z}_t^{**} = \zeta_t \widetilde{Z}_t^*$, where $\Delta\widetilde{M}_{t+1}^* = \mathbf{v}^\top \Delta \mathbf{M}_{t+1}^*$,

$$\begin{aligned} \widetilde{Z}_0^{**} = 1, \qquad \widetilde{Z}_{t+1}^{**} &= \left[1 - \frac{1}{t+1}(1-\gamma^*)\right] \frac{\zeta_{t+1}}{\zeta_t} \zeta_t \widetilde{Z}_t^* + \frac{\zeta_{t+1}}{t+1}\gamma^* \Delta\widetilde{M}_{t+1}^* + \widetilde{O}\left(\frac{\zeta_{t+1}}{t^2}\right) \\ &= \widetilde{Z}_t^{**} + \frac{\zeta_{t+1}}{t+1}\gamma^* \Delta\widetilde{M}_{t+1}^* + \widetilde{O}\left(\frac{\zeta_{t+1}}{t^2}\right). \end{aligned} \tag{S:2}$$

Therefore, we have

$$E[\widetilde{Z}_{t+1}^{**}|\mathscr{F}_t] = \widetilde{Z}_t^{**} + \widetilde{O}(\zeta_{t+1}/t^2).$$

Since $\gamma^* > 0$ and so $\sum_t \zeta_{t+1}/t^2 \sim \sum_t 1/t^{1+\gamma^*} < +\infty$, the process $\widetilde{Z}_t^{**}$ is a non-negative almost (super-)martingale, almost surely convergent toward a finite random variable $\widetilde{Z}_\infty^{**}$ (see Appendix S1.2). Then, using Theorem S1.3, we can prove that $P(\widetilde{Z}_\infty^{**} > 0) = 1$. Indeed, if we define the stochastic process $\mathscr{W} = (\mathscr{W}_t)_{t\geq 0}$, taking values in the interval $[0,1]$, as

$$\begin{aligned} &\mathscr{W}_0 = \widetilde{Z}_0^* \\ &\mathscr{W}_{t+1} = \left(1 - \frac{1}{t+2}\right)\mathscr{W}_t + \frac{1}{t+2} Y_{t+1}, \quad t \geq 0, \end{aligned} \tag{S:3}$$

where $Y_{t+1} = \gamma^* \widetilde{X}_{t+1}^* = \gamma^* \mathbf{v}^\top \mathbf{X}_{t+1}^*$ (that takes values in $[0,1]$, since $\gamma^* < 1$, $X_{t+1,j}^* \in \{0,1\}$ and $\mathbf{v}^\top \mathbf{1} = 1$), then we have

$$|\mathscr{W}_t - \widetilde{Z}_t^*| = O(\ln(t)/t) \to 0$$

and also

$$|\zeta_t \mathscr{W}_t - \widetilde{Z}_t^{**}| = |\zeta_t \mathscr{W}_t - \zeta_t \widetilde{Z}_t^*| = O(\zeta_t \ln(t)/t) = O(\ln(t)/t^{\gamma^*}) \to 0.$$

From Theorem S1.3 applied to $(\mathscr{W}_t)$ with $\delta = \gamma^*$, we get that $\zeta_t \mathscr{W}_t$ converges almost surely to a random variable with values in $(0,+\infty)$. This random variable is obviously also the almost sure limit of $\widetilde{Z}_t^{**}$ and so we can conclude that $P(\widetilde{Z}_\infty^{**} > 0) = 1$.

Furthermore, we can observe that, for each t, we have $|E[\widetilde{Z}_t^{**}] - E[\widetilde{Z}_0^{**}]| \leq \sum_{n=0}^{t-1} |E[\widetilde{Z}_{n+1}^{**}] - E[\widetilde{Z}_n^{**}]| \leq \sum_n |O(\zeta_{n+1}/n^2)|$ and

thus, since the last series is finite, we have $\sup_t E[\widetilde{Z}_t^{**}] < +\infty$. By Fatou's lemma, this fact implies that $\widetilde{Z}_\infty^{**}$ is integrable.

Now, we are ready to prove Lemma S1.2, whose statement and proof is postponed at the end of the present proof. A first consequence of this lemma is that the convergence of $\widetilde{Z}_t^{**}$ to $\widetilde{Z}_\infty^{**}$ is also in mean. Indeed, from (S:2), since $\sup_t E[\widetilde{Z}_t^{**}] < +\infty$ and $(\Delta\widetilde{M}_{t+1}^*)^2 \leq C\sum_{j=1}^N (\Delta M_{t+1,j}^*)^2$, we can obtain

$$E[(\widetilde{Z}_{t+1}^{**})^2] \leq E[(\widetilde{Z}_t^{**})^2] + (\gamma^*)^2 \frac{\zeta_{t+1}^2}{(t+1)^2} CE[V_t^*] + \widetilde{O}(\zeta_{t+1}/t^2),$$

where V_t^* is defined in the statement of Lemma S1.2. Then, we find

$$\begin{aligned} |E[(\widetilde{Z}_t^{**})^2] - E[(\widetilde{Z}_0^{**})^2]| &\leq \sum_{n=0}^{t-1} |E[(\widetilde{Z}_{n+1}^{**})^2] - E[(\widetilde{Z}_n^{**})^2]| \\ &\leq (\gamma^*)^2 \sum_n \frac{\zeta_{n+1}^2}{(n+1)^2} CE[V_n^*] + \sum_n |O(\zeta_{n+1}/n^2)| < +\infty, \end{aligned}$$

where we have used Lemma S1.2 in order to say that the first series is finite. Therefore, we have $\sup_t E[(\widetilde{Z}_t^{**})^2] < +\infty$ and so $(\widetilde{Z}_t^{**})_t$ is uniformly integrable and we can conclude that $\widetilde{Z}_t^{**}$ converges to $\widetilde{Z}_\infty^{**}$ also in mean.

Dynamics of $\mathbf{Z}_{\gamma,t}^{**}$. By multiplying equation (S:1) by ζ_{t+1} we get

$$\begin{aligned} \zeta_{t+1}\mathbf{Z}_{t+1}^* &= \zeta_{t+1}\mathbf{Z}_t^* - \frac{1}{t+1}\zeta_{t+1}(I - \Gamma^\top)\mathbf{Z}_t^* + \frac{1}{t+1}\zeta_{t+1}\Gamma^\top \Delta\mathbf{M}_{t+1}^* + O(\zeta_{t+1}/t^2) \\ \mathbf{Z}_{t+1}^{**} &= \frac{\zeta_{t+1}}{\zeta_t}\mathbf{Z}_t^{**} - \frac{1}{t+1}\frac{\zeta_{t+1}}{\zeta_t}(I - \Gamma^\top)\mathbf{Z}_t^{**} + \frac{\zeta_{t+1}}{t+1}\Gamma^\top \Delta\mathbf{M}_{t+1}^* + O(\zeta_{t+1}/t^2), \end{aligned}$$

where $\mathbf{Z}_t^{**} = \zeta_t \mathbf{Z}_t^*$ and $\Delta\mathbf{M}_t^{**} = \zeta_t \Delta\mathbf{M}_t^*$. Then, using the relation $\zeta_{t+1}/\zeta_t = 1 + (\zeta_{t+1}/\zeta_t)(1-\gamma^*)/(t+1)$ and recalling that $\zeta_{t+1}/\zeta_t = 1 + O(1/t)$, we obtain

$$\begin{aligned} \mathbf{Z}_{t+1}^{**} &= \mathbf{Z}_t^{**} + \frac{\zeta_{t+1}}{\zeta_t}\frac{1-\gamma^*}{t+1}\mathbf{Z}_t^{**} - \frac{1}{t+1}\frac{\zeta_{t+1}}{\zeta_t}(I - \Gamma^\top)\mathbf{Z}_t^{**} + \frac{\zeta_{t+1}}{t+1}\Gamma^\top \Delta\mathbf{M}_{t+1}^* + O(\zeta_{t+1}/t^2) \\ &= \mathbf{Z}_t^{**} - \frac{1}{t+1}(\gamma^* I - \Gamma^\top)\mathbf{Z}_t^{**} + \frac{\zeta_{t+1}}{t+1}\Gamma^\top \Delta\mathbf{M}_{t+1}^* + O(\zeta_{t+1}/t^2). \end{aligned} \tag{S:4}$$

Study of $\mathbf{Z}_{\gamma,t}^{**}$ with $|\gamma| < \gamma^*$. Let $\mathbf{B}_t^{**} = V_\gamma^\top \mathbf{Z}_t^{**}$ and since $\mathbf{Z}_{\gamma,t}^{**} = U_\gamma V_\gamma^\top \mathbf{Z}_t^{**} = U_\gamma \mathbf{B}_t^{**}$, it is enough to prove that $\|\mathbf{B}_t^{**}\|^2$ converges a.s. to zero. To this end, by multiplying equation (S:4) by $V_\gamma^\top$, we have

$$\mathbf{B}_{t+1}^{**} = \left[I - \frac{1}{t+1}(\gamma^* I - J_\gamma^\top)\right]\mathbf{B}_t^{**} + \frac{\zeta_{t+1}}{t+1} J_\gamma^\top V_\gamma^\top \Delta\mathbf{M}_{t+1}^* + O(\zeta_{t+1}/t^2).$$

Without loss of generality, in the following computations we neglect the reminder term $O(\zeta_{t+1}/t^2)$. Then, since for any real matrix A we can write

$$E[\Delta\mathbf{M}_{t+1}^{*\top} A \Delta\mathbf{M}_{t+1}^* | \mathscr{F}_t] = \sum_{j=1}^N a_{jj}^2 E[\Delta M_{j,t+1}^{*2} | \mathscr{F}_t] \leq \max_j a_{jj}^2 V_t^*, \tag{S:5}$$

we have that

$$\begin{aligned} E[\|\mathbf{B}_{t+1}^{**}\|^2 | \mathscr{F}_t] &= \left\| \left[\left(1 - \frac{\gamma^*}{t+1}\right) I + \frac{1}{t+1} J_\gamma\right] \mathbf{B}_t^{**} \right\|^2 + \left(\frac{\zeta_{t+1}^2}{(t+1)^2}\right) \sum_{j=1}^N [\bar{V}_\gamma \bar{J}_\gamma J_\gamma^\top V_\gamma^\top]_{jj}^2 E[\Delta M_{j,t+1}^{*2} | \mathscr{F}_t] \\ &\leq \left(1 - \frac{\gamma^*}{t+1} + \frac{\|J_\gamma\|_{2,2}}{t+1}\right)^2 \|\mathbf{B}_t^{**}\|^2 + \left(\frac{\zeta_{t+1}^2}{(t+1)^2}\right) \max_j \{[\bar{V}_\gamma \bar{J}_\gamma J_\gamma^\top V_\gamma^\top]_{jj}^2\} V_t^*. \end{aligned}$$

Then, regarding the first term, we note that

$$\left(1 - \frac{\gamma^*}{t+1} + \frac{\|J_\gamma\|_{2,2}}{t+1}\right)^2 \leq \left(1 - \frac{\gamma^*}{t+1} + \frac{|\gamma| + \gamma^*}{2(t+1)}\right)^2 = \left(1 - \frac{\gamma^* - |\gamma|}{2(t+1)}\right)^2,$$

and so

$$E[\|\mathbf{B}^{**}_{t+1}\|^2|\mathscr{F}_t] \leq \left(1-\frac{\gamma^*-|\gamma|}{2(t+1)}\right)^2 \|\mathbf{B}^{**}_t\|^2 + C\frac{\zeta^2_{t+1}}{(t+1)^2}V^*_t.$$

Therefore, since $\gamma^* > |\gamma|$ and by Lemma S1.2, the process $\|\mathbf{B}^{**}_t\|^2$ is a non-negative almost supermartingale that converges almost surely. Moreover, by applying the expectation we obtain

$$E[\|\mathbf{B}^{**}_{t+1}\|^2] \leq \left(1-\frac{\gamma^*-|\gamma|}{2(t+1)}\right)^2 E[\|\mathbf{B}^{**}_t\|^2] + C\frac{\zeta^2_{t+1}}{(t+1)^2}E[V^*_t],$$

which, since $\sum_t(\gamma^*-|\gamma|)/(t+1) = +\infty$, by Lemma S1.2 and Lemma S1.6, we can conclude that $\|\mathbf{B}^{**}_t\| \xrightarrow{a.s.} 0$, and hence $\mathbf{B}^{**}_t \xrightarrow{a.s.} \mathbf{0}$.

Study of $\mathbf{Z}^{**}_{\gamma,t}$ with $|\gamma| = \gamma^*$. From the Frobenious-Perron theory, we know that each eigenvalue with maximum modulus is simple: denote by $\mathbf{u}_\gamma$ and $\mathbf{v}_\gamma$ the corresponding eigenvectors. Then, set $b_t = \mathbf{v}_\gamma^\top \mathbf{Z}^{**}_t$ so that, since we have $\mathbf{Z}^{**}_{\gamma,t} = \mathbf{u}_\gamma \mathbf{v}_\gamma^\top \mathbf{Z}^{**}_t = \mathbf{u}_\gamma b_t$, it is enough to prove that $|b_t|$ almost surely converges to zero. To this end, by multiplying equation (S:4) by $\mathbf{v}_\gamma^\top$, we have

$$b^{**}_{t+1} = \left[1-\frac{1}{t+1}(\gamma^*-\gamma)\right] b^{**}_t + \frac{\zeta_{t+1}}{t+1}\gamma \mathbf{v}_\gamma^\top \Delta\mathbf{M}^*_{t+1} + O(\zeta_{t+1}/t^2).$$

Without loss of generality, in the following computations we neglect the reminder term $O(\zeta_{t+1}/t^2)$. Then, using (S:5), we have that

$$\begin{aligned}
E[|b^{**}_{t+1}|^2|\mathscr{F}_t] &= \left|1-\frac{\gamma^*}{t+1}+\frac{\gamma}{t+1}\right|^2 |b^{**}_t|^2 + \left(\frac{\zeta^2_{t+1}}{(t+1)^2}\right)|\gamma|^2 \sum_{j=1}^N |v_j|^2 E[\Delta M^{*2}_{j,t+1}|\mathscr{F}_t] \\
&\leq \left|1-\frac{\gamma^*}{t+1}+\frac{\gamma}{t+1}\right|^2 |b^{**}_t|^2 + \left(\frac{\zeta^2_{t+1}}{(t+1)^2}\right)|\gamma|^2 \max_j\{|v_j|^2\} V^*_t.
\end{aligned}$$

Then, regarding the first term we have that

$$\begin{aligned}
\left|1-\frac{\gamma^*}{t+1}+\frac{\gamma}{t+1}\right|^2 &= \left(1-\frac{\gamma^*}{t+1}+\frac{\mathscr{R}e(\gamma)}{t+1}\right)^2 + \left(\frac{\mathscr{I}m(\gamma)}{t+1}\right)^2 \\
&= 1+\left(\frac{\gamma^*-\mathscr{R}e(\gamma)}{t+1}\right)^2 - 2\left(\frac{\gamma^*-\mathscr{R}e(\gamma)}{t+1}\right) + \left(\frac{\mathscr{I}m(\gamma)}{t+1}\right)^2 \\
&= 1-\left(\frac{2(\gamma^*-\mathscr{R}e(\gamma))}{t+1}\right) + \left(\frac{\gamma^{*2}-2\gamma^*\mathscr{R}e(\gamma)+\mathscr{R}e(\gamma)^2+\mathscr{I}m(\gamma)^2}{(t+1)^2}\right) \\
&= 1-\left(\frac{2(\gamma^*-\mathscr{R}e(\gamma))}{t+1}\right) + \left(\frac{2\gamma^*(\gamma^*-\mathscr{R}e(\gamma))}{(t+1)^2}\right) \\
&= 1-2\left(\frac{1}{t+1}-\frac{\gamma^*}{(t+1)^2}\right)(\gamma^*-\mathscr{R}e(\gamma))
\end{aligned}$$

and so

$$E[|b^{**}_{t+1}|^2|\mathscr{F}_t] \leq \left(1-2\left(\frac{1}{t+1}-\frac{\gamma^*}{(t+1)^2}\right)(\gamma^*-\mathscr{R}e(\gamma))\right)|b^{**}_t|^2 + C\frac{\zeta^2_{t+1}}{(t+1)^2}V^*_t.$$

Therefore, since $\gamma^* > \mathscr{R}e(\gamma)$ and by Lemma S1.2, the process $|b^{**}_t|^2$ is a non-negative almost supermartingale that converges almost surely. Moreover, by applying the expectation, we obtain

$$E[|b^{**}_{t+1}|^2] \leq \left(1-2\left(\frac{1}{t+1}-\frac{\gamma^*}{(t+1)^2}\right)(\gamma^*-\mathscr{R}e(\gamma))\right)E[|b^{**}_t|^2] + C\frac{\zeta^2_{t+1}}{(t+1)^2}E[V^*_t].$$

Since $\sum_t(1/(t+1)-\gamma^*/(t+1)^2) = +\infty$ and by Lemma S1.2 and Lemma S1.6, we can conclude that $|b^{**}_t| \xrightarrow{a.s.} 0$, and hence $b^{**}_t \xrightarrow{a.s.} 0$.

□

Lemma S1.2. *Set $V_t^* = \sum_{j=1}^N E[(\Delta M_{t+1,j}^*)^2 \,|\, \mathscr{F}_t]$. Then, if Γ is irreducible, we have*

$$\sum_t \tfrac{\zeta_{t+1}^2}{(t+1)^2} E[V_t^*] < +\infty \quad \textit{and so} \quad \sum_t \tfrac{\zeta_{t+1}^2}{(t+1)^2} V_t^* < +\infty \textit{ a.s.} \tag{S:6}$$

Proof. First notice that by definition

$$V_t^* = \sum_{j=1}^N E[(\Delta M_{t+1,j}^*)^2 \,|\, \mathscr{F}_t] = \sum_{j=1}^N Z_{j,t}^*(1 - Z_{j,t}^*) \leq \sum_{j=1}^N Z_{j,t}^*.$$

Then, denoting by $v_{\min}$ the minimum element of $\mathbf{v}$, which is strictly positive since $\Gamma^\top$ is irreducible, we have that $\sum_{j=1}^N Z_{j,t}^* \leq \mathbf{v}^\top \mathbf{Z}_t^*/v_{\min} = \widetilde{Z}_t^*/v_{\min}$. Therefore, we have

$$\zeta_t V_t^* \leq \frac{\widetilde{Z}_t^{**}}{v_{\min}}.$$

Therefore, recalling that $\sup_t E[\widetilde{Z}_t^{**}] < +\infty$ and $\frac{\zeta_{t+1}}{(t+1)^2} = O(1/t^{1+\gamma^*})$, we get

$$E\left[\sum_t \frac{\zeta_{t+1}^2}{(t+1)^2} V_t^*\right] = \sum_t \frac{\zeta_{t+1}^2}{(t+1)^2} E[V_t^*] \leq \frac{1}{\gamma^* v_{\min}} \sup_t E[\widetilde{Z}_t^{**}] \sum_t \frac{\zeta_{t+1}}{(t+1)^2} < +\infty.$$

This concludes the proof. □

Proof of Theorem 3.1

Leveraging on Theorem S1.1, we can prove Theorem 3.1. Indeed, by the previous convergence results for $(Z_{t,h}^*)_t$, we have

$$D_{t,h}^* = \sum_{n=1}^t X_{n,h}^* \qquad \text{with } E[X_{t+1,h}^* \,|\, \text{past}] = Z_{t,h}^* \overset{a.s.}{\sim} \frac{\widetilde{Z}_\infty^{**} u_h}{t^{1-\gamma^*}}$$

and so, by Lemma S1.8, we get

$$D_{t,h}^* \overset{a.s.}{\sim} D_{\infty,h}^{**} t^{\gamma^*} \quad \text{with } D_{\infty,h}^{**} = \frac{\widetilde{Z}_\infty^{**} u_h}{\gamma^*}.$$

As a consequence, we obtain

$$\frac{D_{t,h}^*}{D_{t,j}^*} \xrightarrow{a.s.} \frac{D_{\infty,h}^{**}}{D_{\infty,j}^{**}} = \frac{u_h}{u_j}.$$

Proof of Theorem 3.2

Recall from (5) that, for any color c already present in the network at time t, $P_t(h,c) = P(C_{t+1,h} = c|\text{ past})$ denotes the conditional probability that the extraction at time-step $t+1$ from urn h gives the old color c, while $K_t(h,c)$ indicates the number of times the color c has been drawn from urn h until time-step t.

First of all, we observe that, from (5), we have

$$P_t(h,c) = \frac{\sum_{j=1}^N w_{j,h} K_t(j,c) - \gamma_{j^*(c),h}}{\theta_h + t} = \frac{\sum_{n=1}^t \sum_{j=1}^N w_{j,h} \Delta K_n(j,c)}{\theta_h + t} - \frac{\gamma_{j^*(c),h}}{\theta_h + t},$$

where $\Delta K_n(j,c) = K_n(j,c) - K_{n-1}(j,c)$. Notice that $\Delta K_n(j,c)$ takes values in $\{0,1\}$ and $E[\Delta K_{n+1}(j,c)|\text{past}] = P_n(j,c)$. Then, we obtain the following dynamics for $P_t(h,c)$:

$$P_{t+1}(h,c) = (1 - r_{t,h}) P_t(h,c) + r_{t,h} \sum_{j=1}^N w_{j,h} \Delta K_{t+1}(j,c),$$

where $r_{t,h} = 1/(\theta_h + t + 1) = 1/(t+1) + O_h(1/t^2)$. Thus the corresponding vectorial dynamics for $\mathbf{P}_t(c) = (P_t(1,c), \ldots, P_t(N,c))^\top$ is

$$\begin{aligned} \mathbf{P}_{t^*(c)}(c) \neq \mathbf{0}, \qquad \mathbf{P}_{t+1}(c) &= \left(1 - \frac{1}{t+1}\right) \mathbf{P}_t(c) + \frac{1}{t+1} W^\top \Delta \mathbf{K}_{t+1}(c) + O(1/t^2) \\ &= \mathbf{P}_t(c) - \frac{1}{t+1}(I - W^\top)\mathbf{P}_t(c) + \frac{1}{t+1} W^\top \Delta \mathbf{M}_{t+1}(c) + O(1/t^2), \quad \text{for } t \geq t^*(c), \end{aligned} \tag{S:7}$$

where $t^*(c)$ denotes the time-step of the first extraction of c, $\Delta\mathbf{K}_t(c) = (\Delta K_t(1,c),\dots,\Delta K_t(N,c))^\top$, $\Delta\mathbf{M}_{t+1}(c) = \Delta\mathbf{K}_{t+1}(c) - \mathbf{P}_t(c)$ and $O(1/t^2) = (O_1(1/t^2),\dots,O_N(1/t^2))^\top$. We can note that the dynamics of $\mathbf{P}_t(c)$ in (S:7) presents exactly the same form of the dynamics of $\mathbf{Z}_t^*$ in (S:1). Indeed, the only difference lies in the interacting matrix, which is W in (S:7), while was Γ in (S:1). The different conditions on these two matrices, i.e. $W^\top\mathbf{1} = \mathbf{1}$ and $\Gamma^\top\mathbf{1} < \mathbf{1}$, lead through the Frobenious-Perron theory to have different leading eigenvalues, that is $w^* = 1$ for W and $\gamma^* < 1$ for Γ. Then $\mathbf{P}_t(c)$ converges almost surely to a strictly positive random variable, while, as proven above, $\mathbf{Z}_t^*$ converges almost surely to $\mathbf{0}$. To prove the almost sure convergence of $\mathbf{P}_t(c)$, we can apply exactly the same proof of Theorem S1.1 replacing Γ (and the corresponding eigen-structure) by W. In general this simplifies the proof, e.g. $\zeta_t \equiv 1$ and the relation (S:6) (with $\zeta_t \equiv 1$ and $V_t^* = \sum_{j=1}^N E[(\Delta M_{t+1,j}(c))^2 \,|\, \mathscr{F}_t])$ is trivially true. Therefore, since for W we have $\mathbf{u} = \mathbf{1}$, we have

$$\mathbf{P}_t(c) \xrightarrow{a.s.} \widetilde{P}_\infty(c)\mathbf{1},$$

where $\widetilde{P}_\infty(c)$ is a bounded strictly positive random variable. The fact that it is strictly positive comes from Theorem S1.3 with $\delta = w^* = 1$ and $t^*(c)$ as the initial time-step (since $\widetilde{P}_{t^*(c)}(c) > 0$).

Finally, since $K_t(j,c) = \sum_{n=1}^t \Delta K_n(j,c)$ and $E[\Delta K_{n+1}(j,c)|\text{past}] = P_n(j,c) \overset{a.s.}{\sim} \widetilde{P}_\infty(c)$, by Lemma S1.8, we can conclude that

$$K_t(h,c) \overset{a.s.}{\sim} \widetilde{P}_\infty(c)\,t$$

and so the statement of Theorem 3.2 holds true with $K_\infty(c) = \widetilde{P}_\infty(c)$.

S1.1 A general result

Define the stochastic process $\mathscr{W} = (\mathscr{W}_t)_{t\geq 0}$ taking values in the interval $[0,1]$ and following the dynamics

$$\mathscr{W}_{t+1} = \left(1 - \frac{1}{t+2}\right)\mathscr{W}_t + \frac{1}{t+2}Y_{t+1}, \quad t \geq 0, \tag{S:8}$$

where Y_{t+1} takes values in $[0,1]$ and is such that $E[Y_{t+1}\,|\,\text{past}] \overset{a.s.}{\sim} \delta\mathscr{W}_t$ with $0 < \delta \leq 1$, that is $E[Y_{t+1}\,|\,\text{past}] \overset{a.s.}{=} \delta\mathscr{W}_t + R_t$, where $R_t = O(1/t^\beta)$ for some $\beta > (1-\delta)$ [1].

We are going to prove the following result

Theorem S1.3. *Given $\mathscr{W}_0 > 0$, we have that $\mathscr{W}_t$ converges almost surely to 0 as $t^{-(1-\delta)}$, that is $t^{(1-\delta)}\mathscr{W}_t$ converges almost surely to a random variable with values in $(0,+\infty)$.*

First of all, we observe that $\mathscr{W}_0 > 0$ implies $\mathscr{W}_t > 0$ for each t. Moreover, since the assumption on R_t, without loss of generality, we can assume $R_t = 0$. Hence, we note (see[1] for details) that, for each t, the random variable $\mathscr{W}_t$ corresponds to the proportion H_t/s_t of balls of color A inside the urn at time-step t for a two-color urn process where the number of balls of color A (resp. B) added to the urn at time-step t is $U_t^A = \alpha_t Y_t$ (resp. $U_t^B = \alpha_t(1-Y_t)$) with $s_0 = 1$ and $\alpha_t = \frac{1/(t+1)}{\prod_{n=1}^t (1-1/(n+1))} \sim 1$ (and so $s_t = 1/\prod_{n=1}^t (1-1/(k+1)) \sim t$). Note that, if $(\mathscr{F}_t)_t$ is the filtration associated to the urn process, we have

$$E[U_{t+1}^A|\mathscr{F}_t] \overset{a.s.}{\sim} \delta\mathscr{W}_t\,. \tag{S:9}$$

We observe also that, since Y_t takes values in $[0,1]$ and so $Y_t^2 \leq Y_t$, we have

$$E[(U_{t+1}^A)^2|\mathscr{F}_t] \leq \alpha_{t+1}^2 E[Y_{t+1}\,|\,\mathscr{F}_t] \overset{a.s.}{\sim} \delta\mathscr{W}_t\,. \tag{S:10}$$

In the following two lemmas we will show that H_t diverges almost surely to $+\infty$ and $1/H_t = o(t^{-1/\theta})$ for $\theta > 1/\delta$.

Lemma S1.4. *Assuming $\mathscr{W}_0 > 0$, H_t diverges almost surely to $+\infty$*

Proof. Since $H_t = \mathscr{W}_0 + \sum_{n=1}^t U_n^A$, where the random variables U_n^A are positive and uniformly bounded by a constant. By Lemma S1.7, we have $H_t \xrightarrow{a.s.} +\infty$ if and only if $\sum_t E[U_{t+1}^A|\mathscr{F}_t] = +\infty$ almost surely. Therefore, it is enough to observe that this last condition is satisfied when $\mathscr{W}_0 > 0$, because of (S:9) and the fact that $\mathscr{W}_t = H_t/s_t \geq H_0/s_t = \mathscr{W}_0\frac{1}{s_t} \overset{a.s.}{\sim} \mathscr{W}_0/t$. □

Lemma S1.5. *For each $\theta > 1/\delta$, we have $1/H_t = o(t^{-1/\theta})$.*

[1] $R_t = O(1/t^\beta)$ means $|R_t| \leq C/t^\beta$ for a suitable constant C and t large enough.

Proof. We have

$$E\left[\frac{t+1}{H_{t+1}^{\theta}}-\frac{t}{H_t^{\theta}}\,|\,\mathscr{F}_t\right]=E\left[\frac{t+1}{H_t^{\theta}}-\frac{t}{H_t^{\theta}}+\frac{t+1}{H_{t+1}^{\theta}}-\frac{t+1}{H_t^{\theta}}\,|\,\mathscr{F}_t\right]=$$
$$\frac{1}{H_t^{\theta}}+E\left[(t+1)\left(\frac{1}{(H_t+U_{t+1}^A)^{\theta}}-\frac{1}{H_t^{\theta}}\right)|\,\mathscr{F}_t\right]\leq$$
$$\frac{1}{H_t^{\theta}}+tE\left[\left(\frac{1}{(H_t+U_{t+1}^A)^{\theta}}-\frac{1}{H_t^{\theta}}\right)|\,\mathscr{F}_t\right].$$

Let C so that $0\leq U_t^A=\alpha_{t+1}Y_t\leq C$. Using the Taylor expansion of the function $f(x)=1/(a+x)^{\theta}$ (that is $f(x)-f(0)=f'(0)x+\frac{f''(x_0)}{2}x^2$ with $x_0\in(0,x)$) with $a=H_t$ and $x=U_{t+1}^A$, we have eventually (so that $H_t\geq 1$)

$$\frac{1}{(H_t+U_{t+1}^A)^{\theta}}-\frac{1}{H_t^{\theta}}$$
$$\leq -\frac{\theta}{H_t^{\theta+1}}U_{t+1}^A+\frac{\theta(\theta+1)}{H_t^{\theta+2}}(U_{t+1}^A)^2\leq -\frac{\theta}{H_t^{\theta+1}}U_{t+1}^A+\frac{\theta(\theta+1)}{H_t^{\theta+2}}CU_{t+1}^A$$

and so, recalling that $\mathscr{W}_t=H_t/s_t\overset{a.s.}{\sim}H_t/t$, we get

$$E\left[\frac{1}{(H_t+U_{t+1}^A)^{\theta}}-\frac{1}{H_t^{\theta}}|\mathscr{F}_t\right]\leq -\frac{\theta}{H_t^{\theta+1}}\alpha_{t+1}E[Y_{t+1}\,|\,\mathscr{F}_t]\left(1+\frac{(\theta+1)C}{H_t}\right)$$
$$\overset{a.s.}{\sim}-\frac{\theta\delta}{H_t^{\theta}}\frac{1}{t}\left[1+O\left(\frac{1}{H_t}\right)\right].$$

Therefore, we have

$$E\left[\frac{t+1}{H_{t+1}^{\theta}}-\frac{t}{H_t^{\theta}}\,|\,\mathscr{F}_t\right]\leq\frac{1}{H_t^{\theta}t}\left[-(\theta\delta-1)+O\left(\frac{1}{H_t}\right)\right]$$

and so, for $\theta\delta>1$, since $H_t\to+\infty$, we can conclude that the above conditional expectation is eventually negative. This proves that, for each $\theta>1/\delta$, $(t/H_t^{\theta})_t$ is eventually a (positive) super-martingales and so, for each $\theta>1/\delta$, it converges almost surely to a finite random variable. Since $\theta>1/\delta$ is arbitrary, we necessarily have that t/H_t^{θ} converges almost surely to zero. This fact concludes the proof. □

Now we are ready for the proof of the previous theorem.

Proof of Theorem S1.3.
Set $L_t=\ln(H_t/t^{\delta})$, $\Delta_t=E[L_{t+1}-L_t|\mathscr{F}_t]$ and $Q_t=E[(L_{t+1}-L_t)^2|\mathscr{F}_t]$. If we prove that $\sum_t\Delta_t$ and $\sum_t Q_t$ are almost surely convergent, then L_t converges almost surely to a finite random variable (see Lemma S1.9). This fact implies that H_t/t^{δ} converges to a random variable with values in $(0,+\infty)$. The rest of the proof is devoted to verify that $\sum_t|\Delta_t|<+\infty$ and $\sum_t Q_t<+\infty$ almost surely.
To this regard, we note that

$$\Delta_t=E[\ln(H_{t+1})-\ln(H_t)|\mathscr{F}_t]-\delta\left(\ln(t+1)-\ln(t)\right)=$$
$$E[\ln(H_t+U_{t+1}^A)-\ln(H_t)|\mathscr{F}_t]-\delta\ln(1+1/t)=$$
$$E\left[\int_0^{U_{t+1}^A}\frac{1}{H_t+x}dx\right]-\delta\ln(1+1/t).$$

Since $1/(H_t+x)\leq 1/H_t$ and $\ln(1+1/t)\geq 1/t-1/(2t^2)$ for each $x\geq 0$ and each t, the last term of the above equalities is smaller than or equal to

$$\frac{1}{H_t}E[U_{t+1}^A|\mathscr{F}_t]-\frac{\delta}{t}+\frac{\delta}{2t^2}$$

and so, recalling (S:9) and that $\mathscr{W}_t = H_t/s_t \overset{a.s.}{\sim} H_t/t$, it is smaller than or equal to

$$\frac{\alpha_{t+1}E[Y_{t+1}|\mathscr{F}_t]}{H_t} - \frac{\delta}{t} + \frac{\delta}{2t^2} \overset{a.s.}{\sim} \frac{\delta}{t} - \frac{\delta}{t} + \frac{\delta}{2t^2} = O(1/t^2)\,.$$

Therefore $\Delta_t = O(1/t^2)$. Finally, we note that $-\Delta_t = \delta \ln(1+1/t) - \ln(H_{t+1}) + \ln(H_t)$. Using $\ln(1+1/t) \le 1/t$ and $1/(H_t + x) \ge 1/H_t - x/H_t^2$ for each $x \ge 0$ and each t, we find that $-\Delta_t$ is smaller than or equal to

$$\frac{\delta}{t} - \frac{1}{H_t}E[U^A_{t+1}|\mathscr{F}_t] + \frac{1}{2H_t^2}E[(U^A_{t+1})^2|\mathscr{F}_t]$$

and so, recalling (S:9), (S:10) and that $\mathscr{W}_t = H_t/s_t \overset{a.s.}{\sim} H_t/t$, it is smaller than or equal to

$$\frac{\delta}{t} - \frac{\alpha_{t+1}E[Y_{t+1}|\mathscr{F}_t]}{H_t} + \frac{\alpha_{t+1}^2 E[Y_{t+1}|\mathscr{F}_t]}{2H_t^2} \overset{a.s.}{\sim} \frac{\delta}{2tH_t} = O(1/(tH_t)).$$

By the previous Lemma, we have $1/H_t = o(t^{-\eta})$ for some $\eta > 0$ and so $-\Delta_t = O(1/t^{1+\eta})$. Thus, $\sum_t |\Delta_t| < +\infty$ almost surely. Similarly we have

$$\begin{aligned}
&E[(\ln(H_{t+1}) - \ln(H_t) - \delta\ln(t+1) + \delta\ln(t))^2|\mathscr{F}_t] \le \\
&2\left\{E[(\ln(H_{t+1}) - \ln(H_t))^2|\mathscr{F}_t] + \delta(\ln(t+1) - \ln(t))^2\right\} \le \\
&2E\left[\left(\int_0^{U^A_{t+1}} \frac{1}{H_t + x}\,dx\right)^2 \Big|\mathscr{F}_t\right] + 2\delta^2/t^2 \le \\
&2E[(U^A_{t+1}/H_t)^2|\mathscr{F}_t] + O(1/t^2) \le \frac{2}{H_t^2}\alpha_{t+1}^2 E[Y_{t+1}|\mathscr{F}_t] + O(1/t^2) = \\
&O(1/(tH_t)) + O(1/t^2)\,.
\end{aligned}$$

Therefore, we get $Q_t = O(1/t^{1+\eta})$ for some $\eta > 0$ and so $\sum_t Q_t < +\infty$ almost surely. □

S1.2 Non-negative almost supermartingale

Let (Y_n) be an $\mathscr{F}$-adapted sequence of non-negative random variables satisfying

$$E[Y_{n+1}|\mathscr{F}_n] \le (1+\Delta_n)Y_n + R_{1,n} - R_{2,n},$$

where Δ_n, $R_{1,n}$, $R_{2,n}$ are all non-negative sequences of random variables. Then (Y_n) is called non-negative almost supermartingale.

By[2], we know that it almost surely converges on $\{\sum_n \Delta_n < +\infty, \sum_n R_{1,n} < +\infty\}$.

S1.3 Some technical results

For the reader's convenience, we here recall some technical results used in the previous proofs.

Lemma S1.6 ([3, Supplementary material]). *If $a_t \ge 0$, $a_t \le 1$ for t large enough, $\sum_t a_t = +\infty$, $\delta_t \ge 0$, $\sum_t \delta_t < +\infty$, $b > 0$, $y_t \ge 0$ and $y_{t+1} \le (1-a_t)^b y_t + \delta_t$, then $\lim_t y_t = 0$.*

Lemma S1.7 ([4, Theorem 46, p. 40]). *Let $(Y_t)_t$ be a sequence of non-negative random variables, adapted to a filtration $\mathscr{F} = (\mathscr{F}_t)_t$. Then the set $\{\sum_t E[Y_{t+1}|\mathscr{F}_t] < +\infty\}$ is almost surely contained in the set $\{\sum_t Y_t < +\infty\}$. If the random variables Y_t are uniformly bounded by a constant, then these two sets are almost surely equal.*

Lemma S1.8 ([5, Sec. 12.15]). *Let $(Y_t)_t$ be a sequence of Bernoulli random variables, adapted to a filtration $\mathscr{F} = (\mathscr{F}_t)_t$ and such that $Z_t = P(Y_{t+1} = 1\,|\,\mathscr{F}_t)$. Then $\sum_{n=1}^t Y_n / \sum_{n=0}^{t-1} Z_n \xrightarrow{a.s.} 1$.*

Lemma S1.9 ([6, Lemma 3.2]). *Let $(L_n)_n$ be a sequence of random variables, adapted to a filtration $\mathscr{G}_n$. Set $\Delta_n = E[L_{n+1} - L_n|\mathscr{G}_n]$ and $Q_n = E[(L_{n+1} - L_n)^2|\mathscr{G}_n]$. If $\sum_n \Delta_n$ and $\sum_n Q_n$ are almost surely convergent, then $(L_n)_n$ converges almost surely to a finite random variable.*

S2 Heuristics

We here describe an heuristic argument (also employed in[7]), useful in order to detect the rate at which each $D^*_{t,h}$ grows along time in the case of a general matrix Γ.

The dynamics that rules the vectorial process $\mathbf{D}^*_t = (D^*_{t,1}, \dots, D^*_{t,N})^\top$ can be approximated (as $t \to +\infty$) by the linear system of (deterministic) differential equations

$$\dot{\mathbf{d}}^*(t) = \Gamma \frac{\mathbf{d}^*(t)}{t}$$

and hence we can say that $\mathbf{D}^*_t \approx \mathbf{d}^*_t$ for $t \to +\infty$. By the change of variable $t = e^z$, we get

$$\dot{\mathbf{d}}^*(z) = \Gamma \mathbf{d}^*(z)\,,$$

whose general solution is given by $\mathbf{d}^*(z) = e^{\Gamma z}\mathbf{c}$. Now, the term $e^{\Gamma z}$ can be expressed using the canonical Jordan form of the matrix Γ, so that we obtain

$$\mathbf{d}^*(z) = \sum_{k=1}^{r} e^{\gamma_k z} \sum_{i=0}^{p_k-1} z^i \mathbf{c}_i,$$

where $\gamma_1, \dots, \gamma_r$ are the distinct eigenvalues of Γ, $p_1, \dots, p_r$ are the sizes of the corresponding Jordan blocks and $\mathbf{c}_i$ are suitable vectors related to $\mathbf{c}$ and to the generalized eigenvectors of Γ. Indeed, we can write Γ as PJP^{-1}, where J is its canonical Jordan form and P is a suitable invertible matrix of generalized eigenvectors. Therefore, we have $e^{\Gamma z} = Pe^{Jz}P^{-1}$, where e^{Jz} is a block matrix with blocks of the form $e^{J_k z}$ with J_k block in J. On the other hand, if $J_k = \gamma_k I + N_k$ is a generic Jordan block of Γ with size p_k and associated to the eigenvalue γ_k, we have

$$e^{J_k z} = e^{\gamma_k z} e^{N_k z} = e^{\gamma_k z} \sum_{i=0}^{p_k-1} \frac{z^i}{(i-1)!} N_k^i\,.$$

Changing the variable from z to t, we find

$$\mathbf{D}^*_t \approx \mathbf{d}^*(t) = \sum_{k=1}^{r} t^{\gamma_k} \sum_{i=0}^{p_k-1} \ln^i(t)\mathbf{c}_i \tag{S:11}$$

and so the rate at which $D^*_{t,h}$ increases is given by the leading term in the expression of $d^*_h(t)$.

In particular, when Γ is irreducible, the above general formula leads, for each $D^*_{t,h}$, to the same asymptotic behavior t^{γ^*}, with γ^* equal to the leading eigenvalue of Γ (recall that γ^* is simple and so the logarithm term is not present). However, it is important to note that, with this heuristic argument, we can deduce the right rate at which each $D^*_{t,h}$ grows, but we cannot get any information about the limit random variable: we can deduce that, for each h, the quantity $D^*_{t,h}/(u_h t^{\gamma^*})$, where $\mathbf{u}$ is the vector of the relative centrality scores, converges almost surely to a certain random variable (first statement of Theorem 3.1), but we cannot affirm that these limit random variables are all equal and this last fact is fundamental in order to obtain the second statement of Theorem 3.1. Nevertheless, we can affirm that the merit of this heuristics is the fact that, from (S:11), we can get the rate at which each $D^*_{t,h}$ grows for any matrix Γ.

S3 A preliminary idea for the estimation of the interaction in the case $N = 2$

In this section, for the case $N = 2$, we provide a parametric family for the matrix $\Gamma = (\gamma_{j,h})_{j,h=1,2}$ such that its leading eigenvalue γ^* and the ratio $r = u_1/u_2$ of the components of its corresponding left eigenvector coincide with some given values. More precisely, given the values $\gamma^* \in (0,1)$ and $r \in (0,1]$, the matrices

$$\Gamma(x_1,x_2) = \begin{pmatrix} \gamma^*(1-x_1) & \frac{\gamma^*}{r} x_2 I_{(\gamma^* \le r)} + \frac{(1-\gamma^*)}{(1-r)} x_2 I_{(\gamma^* > r)} \\ r\gamma^* x_1 & \gamma^*(1-x_2) I_{(\gamma^* \le r)} + \left[\gamma^* - \frac{(1-\gamma^*)}{(1-r)} r x_2\right] I_{(\gamma^* > r)} \end{pmatrix}, \qquad x_1, x_2 \in (0,1) \tag{S:12}$$

are non-negative, irreducible, such that $\mathbf{1}^\top \Gamma < \mathbf{1}^\top$ and have the leading eigenvalue equal to γ^* and the ratio of the components of the corresponding left eigenvector equal to r. Moreover, we can define a parametric family for the matrix $W = (w_{j,h})_{j,h=1,2}$,

adding other two parameters, as

$$W(x_1,x_2,y_1,y_2) = \Gamma(x_1,x_2) + \Lambda(x_1,x_2,y_1,y_2) \quad \text{where}$$
$$\Lambda(x_1,x_2,y_1,y_2) = \begin{pmatrix} (1-[\Gamma(x_1,x_2)^\top \mathbf{1}]_1)(1-y_1) & (1-[\Gamma(x_1,x_2)^\top \mathbf{1}]_2)y_2 \\ (1-[\Gamma(x_1,x_2)^\top \mathbf{1}]_1)y_1 & (1-[\Gamma(x_1,x_2)^\top \mathbf{1}]_2)(1-y_2) \end{pmatrix}, \qquad y_1, y_2 \in [0,1].$$

Note that the above matrices $W(x_1,x_2,y_1,y_2)$ are non-negative, irreducible and such that $\mathbf{1}^\top W = \mathbf{1}^\top$. The balance condition is satisfied by construction.

Given a data set such that the observed processes exhibit asymptotic behaviors in accordance with the provided theoretical results of the model, the above parametric families for the two interaction matrices Γ and W can be used for performing a Maximum Likelihood Estimation (MLE) procedure. In details:

1) estimate the quantity γ^* as the common slope of the lines in the $\log_{10} - \log_{10}$ plot of the processes $(D^*_{t,h})$, with $h = 1, 2$;

2) estimate the quantity r as $10^{\widehat{u}}$, where $\widehat{u}$ is the difference between the intercepts of the lines in the $\log_{10} - \log_{10}$ plot of the processes $(D^*_{t,h})$, with $h = 1, 2$ (note that, in order to employ the above parametric families of matrices, we need to label the two categories so that the estimated value for r is ≤ 1, i.e. $\widehat{u} \leq 0$);

3) consider the matrices $\Gamma(x_1,x_2)$ and $W(x_1,x_2,y_1,y_2)$ related to the estimated values for γ^* and r;

4) perform a MLE procedure in order to estimate from the data the interaction parameters x_1, x_2, y_1 and y_2 and, possibly, the initial parameters θ_1 and θ_2.

However, in order to get a robust MLE estimation, we may want to reduce the number of parameters by imposing some conditions on them: for instance, we can take θ_1 and θ_2 equal to some given values and restrict to matrices $\Gamma(x_1,x_2)$ and $W(x_1,x_2,y_1,y_2)$ that are symmetric (which means that the interaction mechanism is symmetric, i.e. the influence of $h = 1$ on $h = 2$ is equal to the one of $h = 2$ on $h = 1$). The general formula of the likelihood function that we have to maximize is:

$$\mathcal{L}(\theta_1,\theta_2,x_1,x_2,y_1,y_2;c_{1,1},c_{1,2},\ldots,c_{T,1},c_{2,T}) = \prod_{t=1}^{T-1}\prod_{h=1}^{2}\left(Z^*_{t,h} I_{\{c_{t+1,h}\text{is new}\}} + P_t(h,c) I_{\{c_{t+1,h}\text{is equal to an old item } c\}}\right)$$

where I_E denotes the indicator function of the event E, $Z^*_{t,h}$ and $P_t(h,c)$ are given in (4) and in (5) , respectively, and $(c_{t,1})_{1,\ldots,T}$ and $(c_{t,2})_{1,\ldots,T}$ are the two observed sequences of items (colors/tables) for the two agents (urns/categories) $h = 1, 2$.

We now present a simulation study aimed at highlighting the performance of the estimation procedure obtained by following the steps 1)-4) of the algorithm proposed above. In order to reduce the number of parameters to be estimated, we set $\theta_1 = \theta_2 = 1$ and we impose that both Γ and W must be symmetric. This assumption, combined with the condition $W^\top \mathbf{1} = \mathbf{1}$, implies that Γ and W can be univocally identified by four parameters, e.g. $\gamma_{1,1}$, $\gamma_{1,2}$, $\gamma_{2,2}$, $w_{1,2}$. For each choice of Γ and W, 100 independent innovation processes following the model presented in this work have been generated until the time-step $T = 10^4$. Then, we have applied steps 1)-4) to the data generated by each simulation, so obtaining a set of 100 estimates of γ^*, r, x_1, x_2, y_1 and y_2 which fulfill the symmetric condition, i.e. each one leading to symmetric estimated matrices $\widehat{\Gamma}$ and $\widehat{W}$. The results of this simulation study are collected in Table S1, where the mean values and the standard deviations of the estimated elements are compared with the true ones used for generating the data. Regarding the elements of the two matrices and γ^*, the estimation procedure works very well in all the cases. Regarding r, we can note that the estimated values are "sensitive" to the strenght of the interaction term $\gamma_{1,2}$: the higher the interaction term, the better is the estimation.

In order to complete the picture, we have also checked how the results can be affected by the choice of θ_h and, in particular, if choosing a wrong value of θ_h in the likelihood could considerably worsen the estimation of Γ and W. To this end, we have considered some of the scenarios presented in Table S1 and we have computed the estimates of the elements of Γ and W for two different values of θ_h and, in particular, including the cases when the value of θ_h used to generate the simulated data sets is different from the value of θ_h used to compute the likelihood. The results of this simulation study on the "sensitivity" of the parameter θ_h are collected in Table S2. In general, we can notice that the results seem to be quite robust to the choice of θ_h used in the likelihood. Therefore, the problem of using the "right" θ_h in the likelihood does not seem so important as we could imagine. However, the performance of the estimation procedure does worsen considerably when the data are generated with high values of θ_h. This is probably due to the fact that, when θ_h is large, the asymptotic behaviors of the innovation processes are reached after a number of time-steps which is much larger than $T = 10^4$ used in this simulation study.

In conclusion, the estimation procedure provided in this subsection is only a first step toward the estimation of the interaction between two innovation processes. Additional simulations and analyses are needed. In particular, we need to understand how to test the restrictions on the parameters, for example how to provide a test on the symmetry of the interaction mechanism.

Table S1. Simulation results of the estimation procedure described in steps 1)-4) with $\theta_1 = \theta_2 = 1$ and assuming Γ and W symmetric. Each parameter has been estimated by 100 independent simulated processes generated until time-step $T = 10^4$.
Columns 1-4: elements of the interacting matrices Γ and W used to generate the data.
Columns 5-8: mean values and standard deviations of the elements of the 100 estimated interacting matrices $\widehat{\Gamma}$ and $\widehat{W}$.
Colmuns 9-10: true γ^* and r .
Columns 11-12: mean values and standard deviations of the 100 estimates of $\widehat{\gamma^*}$ and $\widehat{r}$.

$\gamma_{1,1}$	$\gamma_{2,2}$	$\gamma_{1,2}$	$w_{1,2}$	$\widehat{\gamma}_{1,1}$	$\widehat{\gamma}_{2,2}$	$\widehat{\gamma}_{1,2}$	$\widehat{w}_{1,2}$	γ^*	r	$\widehat{\gamma^*}$	$\widehat{r}$
0.10	0.40	0.10	0.50	0.10 (0.07)	0.38 (0.05)	0.14 (0.03)	0.52 (0.03)	0.43	0.30	0.43 (0.04)	0.42 (0.11)
0.10	0.40	0.10	0.25	0.14 (0.08)	0.39 (0.05)	0.12 (0.03)	0.26 (0.02)	0.43	0.30	0.44 (0.05)	0.41 (0.10)
0.25	0.40	0.10	0.50	0.27 (0.06)	0.39 (0.05)	0.12 (0.03)	0.50 (0.03)	0.45	0.50	0.46 (0.04)	0.63 (0.16)
0.25	0.40	0.10	0.25	0.28 (0.06)	0.39 (0.05)	0.11 (0.03)	0.25 (0.02)	0.45	0.50	0.46 (0.04)	0.63 (0.14)
0.10	0.40	0.25	0.50	0.10 (0.05)	0.39 (0.04)	0.26 (0.03)	0.51 (0.03)	0.54	0.57	0.54 (0.03)	0.59 (0.06)
0.10	0.40	0.25	0.25	0.13 (0.05)	0.40 (0.04)	0.25 (0.01)	0.25 (0.01)	0.54	0.57	0.54 (0.03)	0.60 (0.05)
0.25	0.40	0.25	0.50	0.26 (0.04)	0.40 (0.04)	0.25 (0.03)	0.50 (0.03)	0.59	0.74	0.59 (0.03)	0.76 (0.07)
0.25	0.40	0.25	0.25	0.27 (0.04)	0.40 (0.04)	0.24 (0.02)	0.25 (0.02)	0.59	0.74	0.59 (0.02)	0.76 (0.08)
0.10	0.40	0.40	0.50	0.11 (0.04)	0.40 (0.04)	0.40 (0.03)	0.50 (0.03)	0.68	0.69	0.68 (0.02)	0.69 (0.03)
0.25	0.40	0.40	0.50	0.25 (0.02)	0.40 (0.03)	0.40 (0.02)	0.50 (0.02)	0.73	0.83	0.73 (0.02)	0.83 (0.03)

Table S2. Simulation results of the estimation procedure described in steps 1)-4) with $\theta_1 = \theta_2$ and assuming Γ and W symmetric. Each parameter has been estimated by 100 independent simulated processes generated until time-step $T = 10^4$.
Columns 1: value of $\theta_1 = \theta_2 = \theta_{Data}$ used to generate the data.
Columns 2: value of $\theta_1 = \theta_2 = \theta_{Likelihood}$ put in the likelihood function.
Columns 3-6: elements of the interacting matrices Γ and W used to generate the data.
Columns 7-10: mean values and standard deviations of the elements of the 100 estimated interacting matrices $\widehat{\Gamma}$ and $\widehat{W}$.

θ_{Data}	$\theta_{Likelihood}$	$\gamma_{1,1}$	$\gamma_{2,2}$	$\gamma_{1,2}$	$w_{1,2}$	$\widehat{\gamma}_{1,1}$	$\widehat{\gamma}_{2,2}$	$\widehat{\gamma}_{1,2}$	$\widehat{w}_{1,2}$
1	1	0.10	0.40	0.10	0.50	0.10 (0.07)	0.38 (0.05)	0.14 (0.03)	0.52 (0.03)
1	100	0.10	0.40	0.10	0.50	0.09 (0.07)	0.38 (0.05)	0.13 (0.03)	0.51 (0.03)
100	1	0.10	0.40	0.10	0.50	0.22 (0.02)	0.41 (0.02)	0.18 (0.01)	0.5 (0.01)
100	100	0.10	0.40	0.10	0.50	0.19 (0.02)	0.40 (0.02)	0.19 (0.01)	0.51 (0.01)
1	1	0.10	0.40	0.10	0.25	0.14 (0.08)	0.39 (0.05)	0.12 (0.03)	0.26 (0.02)
1	100	0.10	0.40	0.10	0.25	0.11 (0.09)	0.38 (0.05)	0.13 (0.03)	0.26 (0.02)
100	1	0.10	0.40	0.10	0.25	0.28 (0.02)	0.43 (0.01)	0.14 (0.01)	0.26 (0.01)
100	100	0.10	0.40	0.10	0.25	0.27 (0.02)	0.43 (0.01)	0.15 (0.01)	0.26 (0.01)
1	1	0.25	0.40	0.40	0.50	0.25 (0.02)	0.40 (0.03)	0.40 (0.02)	0.50 (0.02)
1	100	0.25	0.40	0.40	0.50	0.25 (0.03)	0.40 (0.03)	0.40 (0.02)	0.50 (0.02)
100	1	0.25	0.40	0.40	0.50	0.29 (0.02)	0.42 (0.02)	0.41 (0.01)	0.50 (0.01)
100	100	0.25	0.40	0.40	0.50	0.29 (0.02)	0.42 (0.02)	0.41 (0.01)	0.50 (0.01)

References

1. Aletti, G., Crimaldi, I. & Ghiglietti, A. Networks of reinforced stochastic processes: estimation of the probability of asymptotic polarization. arXiv:2212.07687, DOI: 10.48550/arXiv.2212.07687 (2022).

2. Robbins, H. & Siegmund, D. A convergence theorem for non negative almost supermartingales and some applications. In Rustagi, J. S. (ed.) *Optimizing Methods in Statistics*, 233–257, DOI: 10.1016/B978-0-12-604550-5.50015-8 (Academic Press, New York, 1971).

3. Aletti, G., Crimaldi, I. & Ghiglietti, A. Networks of reinforced stochastic processes: a complete description of the first-order asymptotics. arXiv:2206.07514, DOI: 10.48550/arXiv.2206.07514 (2022).

4. Dellacherie, C. & Meyer, P.-A. *Probabilities and potential. B*, vol. 72 of *North-Holland Mathematics Studies* (North-Holland Publishing Co., Amsterdam, 1982). Theory of martingales, Translated from the French by J. P. Wilson.

5. Williams, D. *Probability with Martingales* (Cambridge University Press, 1991).

6. Pemantle, R. & Volkov, S. Vertex-reinforced random walk on **Z** has finite range. *Ann. Probab.* **27**, 1368–1388, DOI: 10.1214/aop/1022677452 (1999).

7. Iacopini, I., Di Bona, G., Ubaldi, E., Loreto, V. & Latora, V. Interacting discovery processes on complex networks. *Phys. Rev. Lett.* **125**, 248301, DOI: 10.1103/PhysRevLett.125.248301 (2020).

Author contributions

All the authors contributed equally to the present work.
