# Supplementary material for: Interacting innovation processes
Source: Sci Rep. 2023 Oct 11;13:17187. doi: 10.1038/s41598-023-43967-1 (PMC10567777; doi:10.1038/s41598-023-43967-1)
Supplement: Supplementary file 2 — Supplementary Information 2. [file 41598_2023_43967_MOESM2_ESM.pdf]

Auxiliary analysis for Interacting Innovation processes

Giacomo Aletti[1], Irene Crimaldi[2], and Andrea Ghiglietti[3*]

[1]ADAMSS Center, Università degli Studi di Milano, Milan, Italy
[2]IMT School for Advanced Studies Lucca, Lucca, Italy
[3]Università degli Studi di Milano-Bicocca, Milan, Italy
[*]Corresponding author: andrea.ghiglietti@unimib.it

ABSTRACT

In the present supplementary file we provide some supplementary analysis.

T1 Behaviours observed in modified datasets

The aim of this section is to investigate which behaviours can be observed if we artificially modify the real data sets, in order to show that the behaviors predicted by our theoretical results can only be observed by the original data sets. This analysis highlights how the features described in Section 3.2 and presented by Reddit and Gutenberg data are actually the result of a complex interaction phenomenon and are not just some statistical inevitability that would be observed by any dataset. In particular, we have considered the following two scenarios:

(i) the categories of the pair of customers that enters the restaurant at each time-point are randomly assigned, i.e. randomly assign the genre in Gutenberg data or the sentiment in Reddit data;

(ii) the pairs of customers that enters the restaurant at each time-point are randomly shuffled so changing the original chronological order, i.e. shuffle words in Gutenberg data or the commnets in Reddit data.

In Scenario (i) the categories are meaningless as we have assign them completely at random. As a consequence, the innovation processes of the two categories should be extremely similar. In other words, the behaviour of $(D^*_{t,h})$ and $(D_{t,h})$ should present no difference between the categories $h = 1, 2$, as highlighted by following Figure T1 below, where the pair of lines coincide.

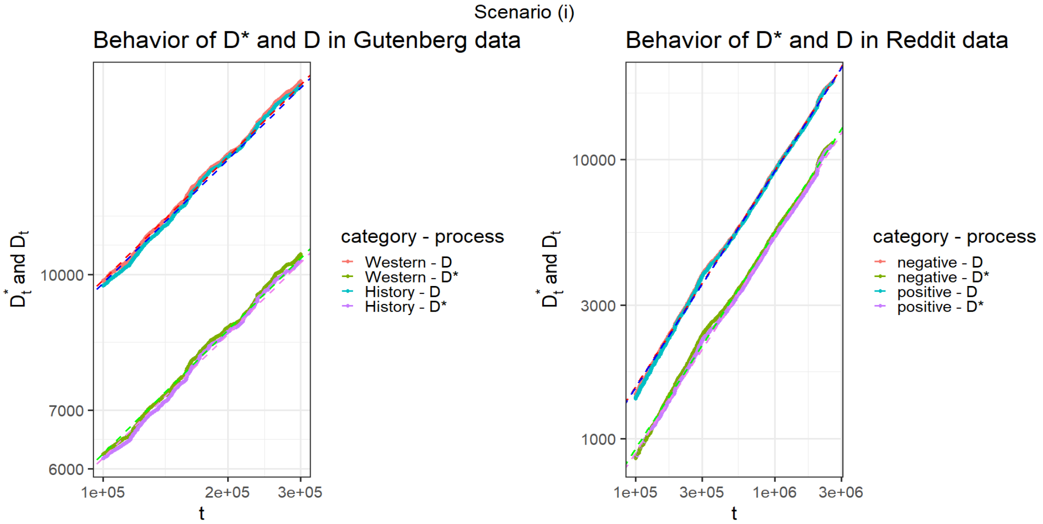

Figure T1. Scenario (i): shuffle of the categories. Linear behaviour of $(D^*_{t,h})$ and $(D_{t,h})$ along time, for $h = 1, 2$, in $\log_{10} - \log_{10}$ scale. The dashed lines are obtained by a least square interpolation with the same common slope for all four lines.

In Scenario (ii) instead, the categories are not mixed up but the temporal autocorrelation of the innovation processes is

removed and so the interaction between the two categories that was based on the temporal dependence is completely broken. This aspect is also highlighted in Figure T2 below, where the lines of $(D^*_{t,h})$ and $(D_{t,h})$ in $\log_{10} - \log_{10}$ scale present now different slopes for the two categories $h = 1, 2$.

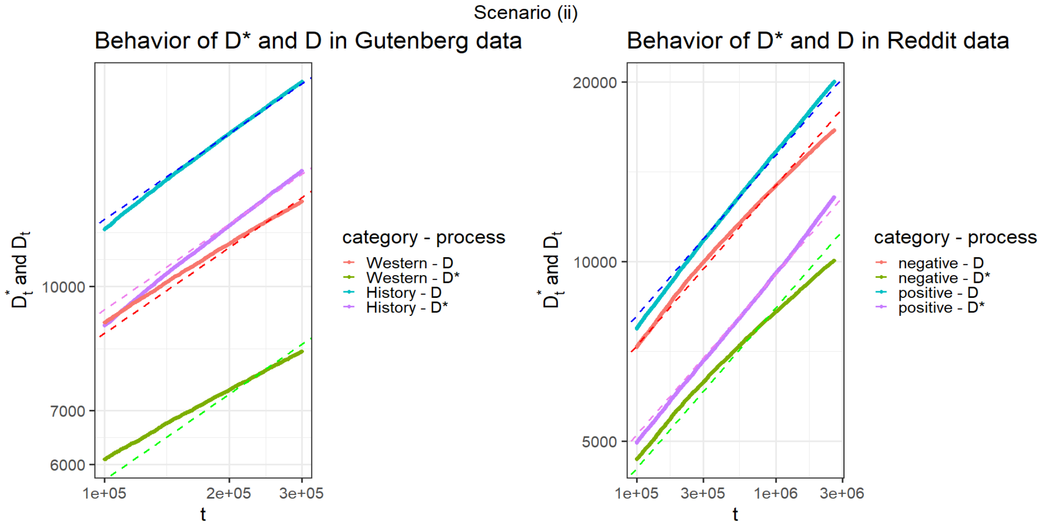

Figure T2. Scenario (ii): shuffle of the time-points. Linear behaviour of $(D^*_{t,h})$ and $(D_{t,h})$ along time, for $h = 1, 2$, in $\log_{10} - \log_{10}$ scale. The dashed lines are obtained by a least square interpolation with the same common slope for all four lines.

T2 Study of the fluctuations of $D_1^*(t)/D_2^*(t)$

The aim of this section is to show that, in real data, the ratio $r = D_1^*(t)/D_2^*(t)$ asymptotically does not fluctuate in different realizations of the process. This property is naturally true theoretically, as it follows from Theorem 3.1, but here we also check it in the two datasets Gutenberg and Reddit. First, let us recall that, in the main paper, we have estimated the value r and made the plots (see Figure 2 for Reddit and Figure 5 for Gutenberg in the article) of the quantity $\log_{10}(D^*_{t,1}/D^*_{t,2})$ along time, in order to appreciate that it asymptotically approaches r. Then, here below we have performed some further analyses on the asymptotic behaviour of the fluctuations of the process $\log_{10}(D^*_{t,1}/D^*_{t,2})$ in the real data sets, regardless of its specific limit. In particular, our aim is to show that the variance of this process converges to zero, i.e. different realizations of the process present values always closer to each others as the time increases to infinity. To this purpose, since each real data set actually represents a single realization of the process, we have decided to resample the original data sets and so investigate the variability of the process on these new samples. Specifically, we have adopted two different resampling procedures:

- the original sequence is randomly divided in n subsequences of the same length. Larger is the number of sequences n generated and lower is the number of time-points in each of them. In this case, any of the original data belongs to only one of the n subsequences generated. Results are collected below in Figure T3 and Figure T4;

- multiple sequences are generated by randomly sampling each original data with a given probability p. Larger is the value of p and larer will be the average number of time-points in each of sequence. In this case, a given original data could belong to more than one of the subsequences generated. Results are collected below in Figure T5 and Figure T6.

. In all cases we can appreciate how the fluctuation of the ratio $r = D_1^*(t)/D_2^*(t)$ seems to vanish as the time goes to infinity.

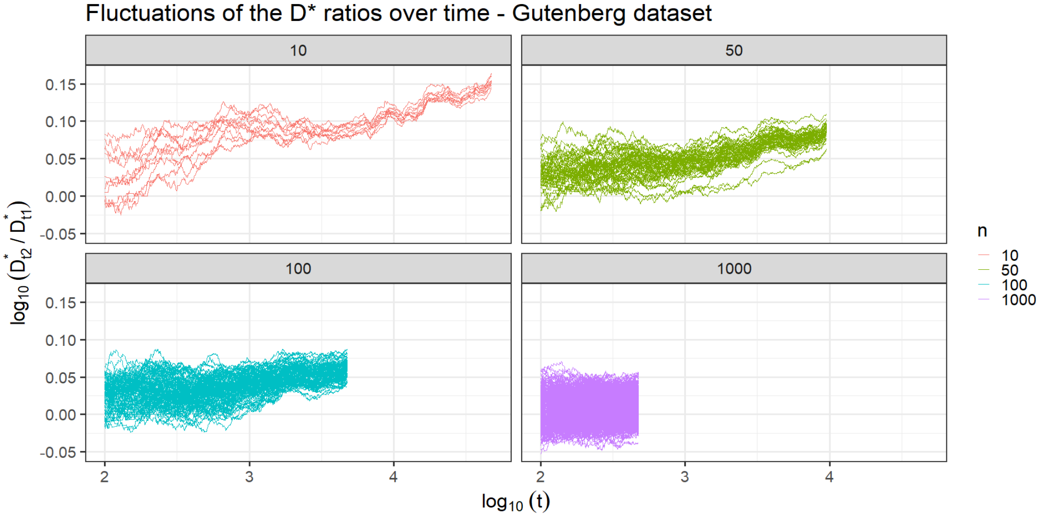

Figure T3. n subsequences of the Gutenberg dataset generated by randomly dividing the original sequence.

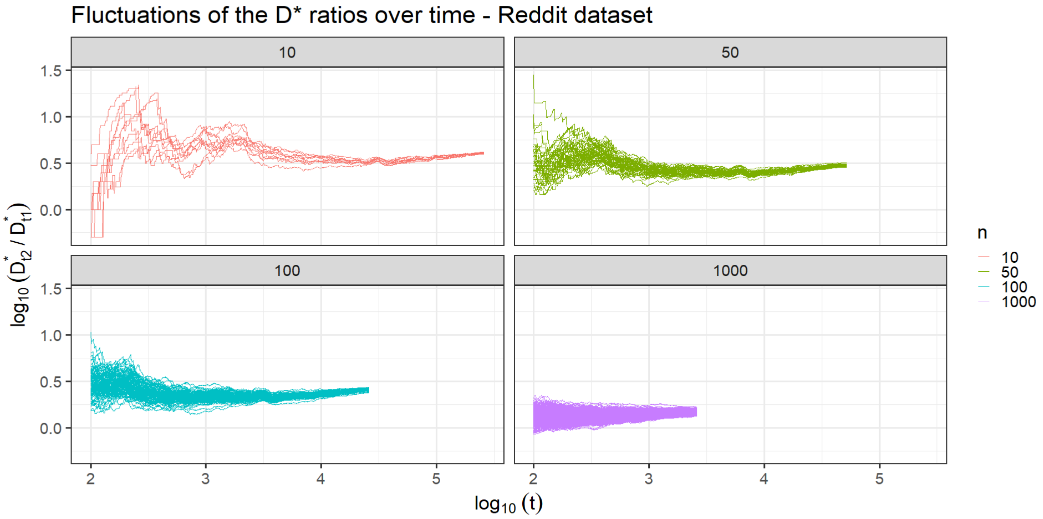

Figure T4. n subsequences of the Reddit dataset generated by randomly dividing the original sequence.

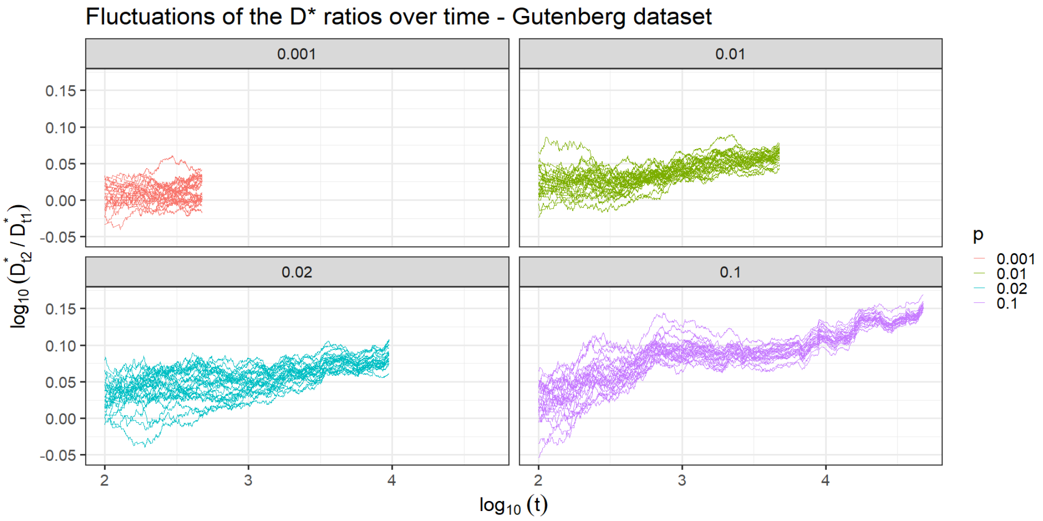

Figure T5. 25 subsequences of the Gutenberg dataset generated by randomly sampling each original data with a given probability p.

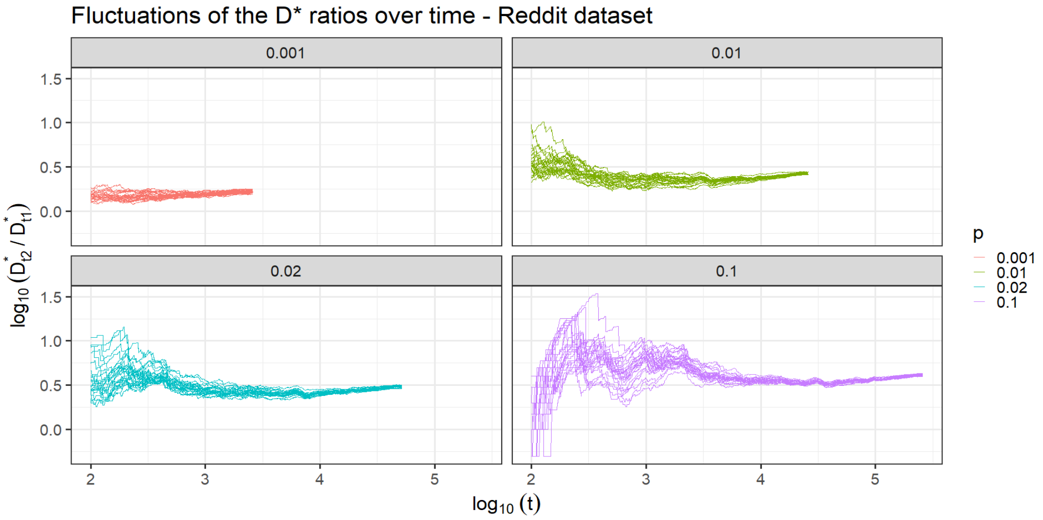

Figure T6. 25 subsequences of the Reddit dataset generated by randomly sampling each original data with a given probability p.

Author contributions

All the authors contributed equally to the present work.
